# Deletion of *Wt1* during early gonadogenesis leads to differences of sex development in male and female adult mice

**DOI:** 10.1371/journal.pgen.1010240

**Published:** 2022-06-15

**Authors:** Alejo Torres-Cano, Rosa Portella-Fortuny, Claudia Müller-Sánchez, Sonia Porras-Marfil, Marina Ramiro-Pareta, You-Ying Chau, Manuel Reina, Francesc X. Soriano, Ofelia M. Martínez-Estrada

**Affiliations:** 1 Celltec-UB, Department of Cell Biology, Physiology and Immunology, Faculty of Biology, University of Barcelona, Barcelona, Spain; 2 Institute of Biomedicine (IBUB), University of Barcelona, Barcelona, Spain; 3 Centre for Cardiovascular Science, Queen’s Medical Research Institute, University of Edinburgh, United Kingdom; 4 Institut de Neurociències, Universitat de Barcelona, Barcelona, Spain; Seattle Children’s Research Institute, UNITED STATES

## Abstract

Assessing the role of the WT1 transcription factor (*WT1)* during early gonad differentiation and its impact on adult sex development has been difficult due to the complete gonadal agenesis and embryonic lethality exhibited by *Wt1KO* mouse models. Here, we generated *Wt1*^*LoxP/GFP*^*;Wt1*^*Cre*^ mice, the first *Wt1KO* mouse model that reaches adulthood with a dramatically reduced *Wt1* expression during early gonadogenesis. *Wt1*^*LoxP/GFP*^*;Wt1*^*Cre*^ mice lacked mature gonads and displayed genital tracts containing both male and female genital structures and ambiguous genitalia. We found that WT1 is necessary for the activation of both male and female sex-determining pathways, as embryonic mutant gonads failed to upregulate the expression of the genes specific for each genetic programme. The gonads of *Wt1*^*LoxP/GFP*^*;Wt1*^*Cre*^ mice showed a lack of production of Sertoli and pre-granulosa cells and a reduced number of germ cells. NR5A1 and the steroidogenic genes expression was modulated differently in XY and XX *Wt1*^*LoxP/GFP*^*;Wt1*^*Cre*^ gonads, explaining the mutant phenotypes. Further studies of the XX *Wt1*^*LoxP/GFP*^*;Wt1*^*Cre*^ gonads revealed that deletion of WT1 at an early stage impaired the differentiation of several cell types including somatic cells and the ovarian epithelium. Through the characterisation of this *Wt1KO* mouse model, we show that the deletion of *Wt1* during early gonadogenesis produces dramatic defects in adult sex development.

## Author summary

The WT1 transcription factor (WT1) is a protein expressed during gonad development. *WT1* mutations have been reported in several human conditions in which patients present a variable range of genital malformations varying from ambiguous external genitalia to gonadal dysgenesis. Mouse models in which *Wt1* has been deleted indicate that WT1 has a critical role in early gonadogenesis. However, assessing the role of this protein in early gonad formation and its impact on adult sex development has been difficult due to the complete gonadal agenesis or embryonic lethality observed in these mouse models. Here, we describe a new genetically engineered mouse model in which *Wt1* expression is deleted from an early stage in gonad formation. The analyses of these mice revealed the importance of *Wt1* for early gonad differentiation and the impact of its early deletion on the formation of the adult reproductive system. Adult mutant mice lacked mature gonads, with both XX and XY mutants displaying genital tracts containing both male and female structures as well as ambiguous external genitalia. Notably, mutant gonads remained in an undifferentiated stage, indicating that WT1 is important for the differentiation of different populations of progenitor cells.

## Introduction

Disorders/differences of sex development (DSDs) are congenital conditions in which the development of chromosomal, gonadal or anatomical sex is atypical [1]. During the last years, considerable efforts have been made to understand the genetic factors involved in DSDs. However, many patients with DSDs cannot be provided with an accurate diagnosis as the aetiology of many DSDs remains unknown [2].

Most of the knowledge about the complex process of sex development in mammals comes from the analysis of mouse models in which the expression of key genes involved in sex development has been modified [3]. By embryonic day (E) 10.5 in mice, bipotential gonads arise from the genital ridges, a structure derived from the intermediate mesoderm [3]. Its formation begins with the proliferation of coelomic epithelial cells that give rise to the somatic gonadal precursors [4]. The molecular events that determine gonadal fate in mice occur at around E11.5–12.0, when, following changes in gene expression, the bipotential gonad begins a specific developmental programme that will generate either a testis or an ovary [3]. Sex determination of bipotential gonads and the subsequent testicular or ovarian differentiation are critical steps not only in establishing the sex of an individual, but also in maintaining their reproduction [3].

The Wilms tumor 1 homolog (*Wt1*) encodes a zinc finger protein whose best-known function is as a transcription factor. WT1 can act as either a transcriptional activator or a repressor depending on its binding partners in a tissue or organ [5]. During gonad formation, WT1 is initially expressed in the intermediate mesoderm and, later on, in the coelomic epithelium of the genital ridge and the somatic cells of the bipotential gonad [6,7]. Following sex determination, WT1 is also expressed in Sertoli cells and granulosa cells [8]. *WT1* mutations have been reported in several human conditions including Denys-Drash, Frasier and Meacham syndromes [5]. In all these syndromes, the affected XY children present a variable range of genital malformations varying from ambiguous or female external genitalia to gonadal dysgenesis [5]. Recently, XX patients carrying *WT1* mutations have been identified among 46,XX *SRY*-negative individuals with different features of DSDs, including testicular DSD (TDSD) or ovotesticular DSD and atypical external genitalia [9–11].

In the last ten years, new mouse genetic tools have been generated and used in several laboratories interested in different aspects of WT1 biology [12–17]. To circumvent the embryonic lethality of the germline *Wt1KO* mouse model and to uncover new functions of *Wt1* in development and adult homeostasis, mice carrying a *loxP*-flanked allele of the *Wt1* gene have been generated and bred with different Cre transgenic animals [12,13,17]. In this study, we generated a new mouse model of the *Wt1* gene, the *Wt1*^*LoxP/GFP*^*;Wt1*^*Cre*^ mice that display a strong reduction of *Wt1* expression during early gonadogenesis. This new *Wt1KO* mouse model is the first model that reaches adulthood with a dramatically reduced *Wt1* expression at an early stage of gonadal development. Interestingly, the analysis of adult *Wt1*^*LoxP/GFP*^*;Wt1*^*Cre*^ mice revealed the presence of ambiguous external genitalia that correlated with the absence of mature gonads, as well as the occurrence of genital tracts containing both male and female structures. We found that WT1 is necessary for the activation of both male and female sex-determining pathways, as mutant gonads failed to upregulate the expression of the genes specific for each programme, resulting in dramatic defects in the formation of somatic and germ cells.

## Results

### *Wt1*^*Cre*^ transgene efficiently recombines in the embryonic gonad

**Fig 1:**
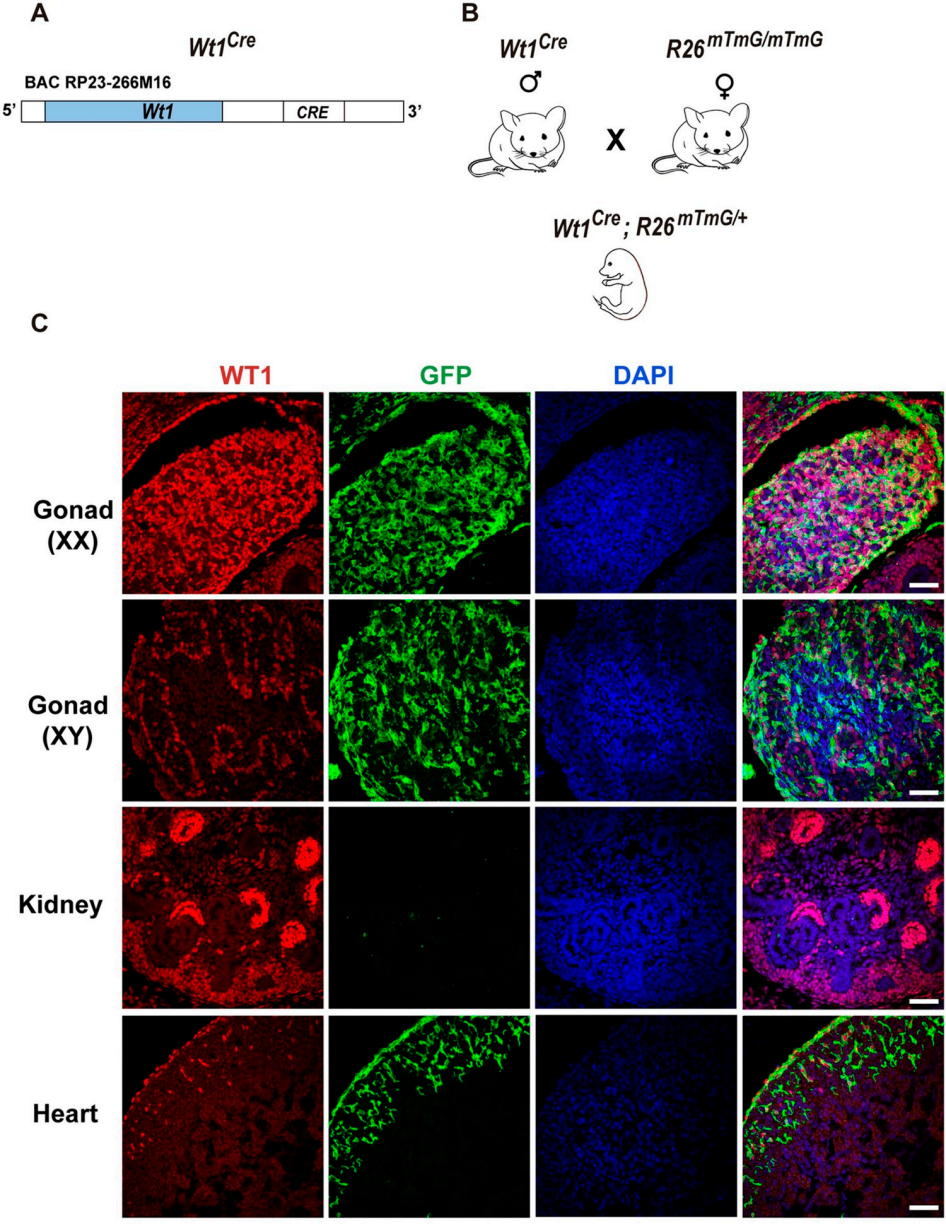
The *Wt1*^*Cre*^ transgene recombines efficiently in the embryonic gonads. (A) Schematic representation of the modified BAC construct used to generate the *Wt1*^*Cre*^ transgenic line. (B) Breeding scheme to generate *Wt1*^*Cre*^*;R26*^*mTmG/+*^ embryonic mice. (C) Immunofluorescence staining for GFP (green) and WT1 (red), using organ sections from *Wt1*^*Cre*^*;R26*^*mTmG/+*^ E14.5 mice. Consistent with the WT1 expression, robust Cre-mediated reporter expression is observed in the XX and XY gonads and in the heart. Note the absence of Cre activity (GFP-positive cells) in the kidneys despite the abundant levels of WT1 endogenous protein. Representative immunostaining images of at least three embryos are shown. Scale bars: 50 μm.

One of the earliest markers that define the somatic cell lineages in the gonads is WT1 [18–20]. We decided to study the activity of *Wt1*^*Cre*^ (Tg(Wt1-cre)#Jbeb) in the gonads, a BAC transgenic mouse line that has been extensively used in the cardiovascular field, but whose expression in the embryonic gonads has not been analysed previously (Fig 1A) [16,21]. This *Wt1*^*Cre*^ line was generated by an insertion of the *IRES-EGFP-Cre* cassette downstream of the translation stop site of the *Wt1* gene. The EGFP protein is undetectable in the transgenic line when assessing with organ section staining or FACS analysis [16]. To monitor *Wt1*^*Cre*^ activity, *Wt1*^*Cre/+*^ *(Wt1*^*Cre*^*)* mice were crossed with the reporter *R26*^*mTmG/mTmG*^ mice (Fig 1B). In the *Wt1*^*Cre*^*;R26*^*mTmG/+*^ mouse model, Tomato is expressed ubiquitously. After Cre-mediated *loxP* recombination, GFP membrane expression is observed. Consistent with WT1 expression, immunostaining analysis of GFP expression in the developing gonads revealed robust Cre-mediated reporter expression in *Wt1*^*Cre*^*;R26*^*mTmG/+*^ mice (Figs 1C and S1A). At E14.5, most of the lineage-labelled *Wt1*^*Cre*^ cells (GFP-positive) were positive for WT1 (Fig 1C). Additionally, double immunostaining with antibodies against GFP and the germ cell marker DDX4 demonstrated the absence of recombination in the DDX4-positive germ cells (S1B Fig). Surprisingly, although endogenous WT1 protein is abundantly expressed in the embryonic kidney, almost no GFP-positive cells were detected in this organ, with the exception of a few mesothelial cells surrounding its surface (Figs 1C and S1A). WT1 is expressed in the urogenital ridge that gives rise to the gonads and the adrenal cortex [7]. *Wt1*^*Cre*^*;R26*^*mTmG/+*^ mice constitute a lineage tracing model, which means that all WT1 and WT1-derived cells should be GFP-positive. However, GFP-positive cells were not observed in the adrenal gland of *Wt1*^*Cre*^*;R26*^*mTmG/+*^ mice at E14.5 (S1A Fig). To further verify the GFP immunostaining data with a more quantitative technique, we analysed the percentage of GFP-positive cells in the digested kidneys of *Wt1*^*Cre*^*;R26*^*mTmG/+*^ mice using fluorescence-activated cell sorting (FACS) (S2A and S2B Fig). We also included in our analysis embryonic kidneys from *Wt1*^*GFP/+*^ mice, a reporter mouse model that has been well characterised in studies on kidney development [22,23]. Our results from the FACS analysis supported the immunostaining data. The percentage of GFP-positive cells in the digested kidneys of *Wt1*^*Cre*^*;R26*^*mTmG/+*^ mice was 0.55+/-0.13% in comparison to the 42.75 +/-4.38% (*P < 0.05, Student’s *t*-test) found in those of *Wt1*^*GFP/+*^ mice (S2A and S2B Fig).

Altogether, these results demonstrate that the *Wt1*^*Cre*^ transgene recombines in the embryonic gonad, but its efficiency of recombination is very low in the kidneys and adrenal gland.

### A new mouse model of *Wt1* to study its loss-of-function effect during early gonadogenesis on adult sex development

**Fig 2:**
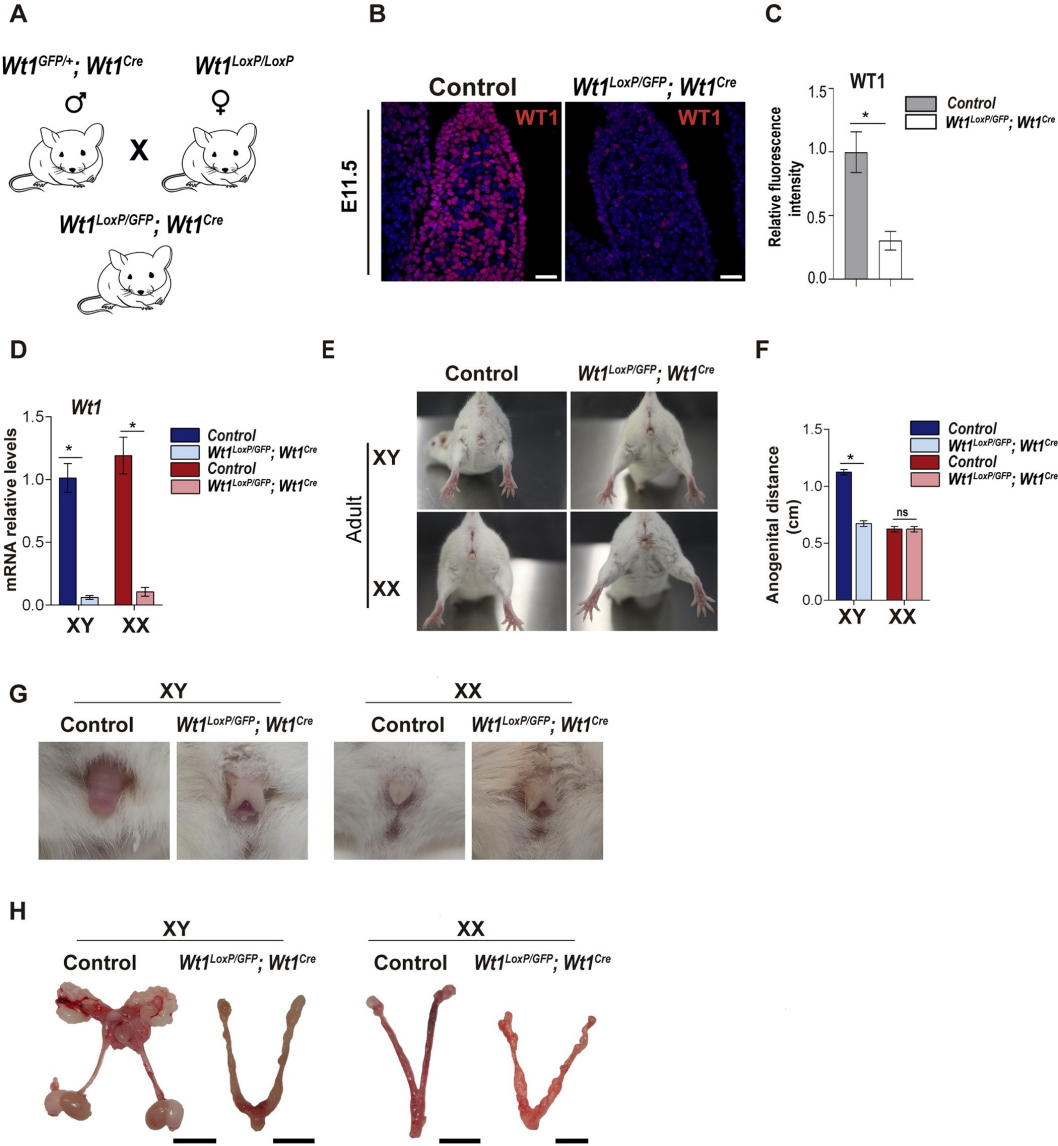
*Wt1*^*LoxP/GFP*^*;Wt1*^*Cre*^ mice, a new *Wt1KO* during early gonadogenesis. (A) Schematic representation of the breeding scheme to generate *Wt1*^*LoxP/GFP*^*;Wt1*^*Cre*^ mice. (B) Immunofluorescence staining for WT1 (red) and nuclear DAPI staining (blue), using sections from the gonads of control and *Wt1*^*LoxP/GFP*^*;Wt1*^*Cre*^ E11.5 mice. (C) Quantification of the WT1 fluorescence intensity in gonads from control and *Wt1*^*LoxP/GFP*^*;Wt1*^*Cre*^ E11.5 mice. Values represent the mean ± s.e.m. (n = 3). *P < 0.05, Student’s *t*-test. (D) qRT-PCR analysis of *Wt1* expression in E12.5 gonads from control and *Wt1*^*LoxP/GFP*^*;Wt1*^*Cre*^ mice. A dramatic downregulation of *Wt1* expression is observed in the gonads of *Wt1*^*LoxP/GFP*^*;Wt1*^*Cre*^ mice. Values represent the mean ± s.e.m. (n = 3–4). *P < 0.05, two-way ANOVA followed by Tukey’s post-hoc test. (E) Images of the external anogenital region of XX and XY control and *Wt1*^*LoxP/GFP*^*;Wt1*^*Cre*^ adult mice. (F) Quantification of the anogenital distance in control and *Wt1*^*LoxP/GFP*^*;Wt1*^*Cre*^ mice. Values represent the mean ± s.e.m. (n = 4). *P < 0.05, two-way ANOVA followed by Tukey’s post-hoc test. (G) Images of the external genitalia of XX and XY control and *Wt1*^*LoxP/GFP*^*;Wt1*^*Cre*^ adult mice. Both XY and XX *Wt1*^*LoxP/GFP*^*;Wt1*^*Cre*^ mice have structures that resemble a micropenis. (H) Gonads and the internal reproductive tracts of XX and XY control and *Wt1*^*LoxP/GFP*^*;Wt1*^*Cre*^ mice. Representative images from a minimum of three each of XX and XY control and mutant mice are shown. Scale bars: 25 μm in B and 1 cm in H.

Next, we decided to estimate whether *Wt1*^*Cre*^ was already active during the earlier stages of gonadogenesis. GFP immunostaining demonstrated very efficient recombination in the gonads of *Wt1*^*Cre*^*;R26*^*mTmG/+*^ mice at E11.5 (S3 Fig). After characterising the efficiency of *Wt1*^*Cre*^ in driving Cre-mediated recombination in the early embryonic gonads, we decided to generate a new mouse model of the *Wt1* gene, the *Wt1*^*LoxP/GFP*^*;Wt1*^*Cre*^ model. These mice were generated by breeding females homozygous for the floxed allele of *Wt1* in exon 1 (*Wt1*^*LoxP/ LoxP*^*)* with males heterozygous for a GFP knock-in of *Wt1* also in exon 1 and *Wt1*^*Cre*^ (*Wt1*^*GFP/+*^*;Wt1*^*Cre*^) (Fig 2A) [13,14,16]. As a result of this breeding scheme, in *Wt1*^*LoxP/GFP*^*;Wt1*^*Cre*^ mice, one copy of the *Wt1* allele is flanked by loxP sites, whereas the other copy has a GFP knock-in in exon 1, which disrupts WT1 expression. Immunostaining analysis of WT1 expression in the embryonic gonads at E11.5 revealed a dramatic reduction in WT1 protein expression in the *Wt1*^*LoxP/GFP*^*;Wt1*^*Cre*^ mice, suggesting that *Wt1*^*Cre*^ was active from the earlier stages of gonad development (Fig 2B and 2C). The immunostaining results were confirmed by the quantitative real-time PCR (qRT-PCR) analysis of *Wt1* expression at E12.5 (Fig 2D). However, the *Wt1* levels were unaltered in the embryonic kidneys of *Wt1*^*LoxP/GFP*^*;Wt1*^*Cre*^ mice, which correlated with the absence of recombination of *Wt1*^*Cre*^ in this organ (Figs 1C, S1 and S2B–S2D). The initial characterisation of the germline *Wt1KO* mouse model indicated that WT1 has a critical role in early gonadogenesis [20]. However, assessing the role of WT1 in bipotential gonads and its impact on adult sex development has been difficult due to the complete gonadal agenesis exhibited by the conventional *Wt1KO* mice and the embryonic lethality observed in *Wt1KO* mouse models [20,24]. The genotyping of the adult mice of our model revealed a sub-Mendelian distribution of the *Wt1*^*LoxP/GFP*^*;Wt1*^*Cre*^ mice. The observed lethality of *Wt1*^*LoxP/GFP*^*;Wt1*^*Cre*^ mice probably corresponds to the late embryonic stages since the proportion of this genotype was not reduced when embryos were examined on days E14.5 (**S1 Table**). As *Wt1*^*Cre*^ is expressed in the heart, we suspect that WT1 deletion in the embryonic heart could be the main cause of lethality in some mutants. A very interesting finding was that all the mutant adult *Wt1*^*LoxP/GFP*^*;Wt1*^*Cre*^ mice were phenotypically female externally. Based on these results, we suspected a feminisation of XY *Wt1*^*LoxP/GFP*^*;Wt1*^*Cre*^ mice, which was confirmed by sex genotyping. The external genitalia in XY *Wt1*^*LoxP/GFP*^*;Wt1*^*Cre*^ mice were feminised, making all the XY *Wt1*^*LoxP/GFP*^*;Wt1*^*Cre*^ mice indistinguishable externally from the control XX mice with their short anogenital distances (Fig 2E and 2F). Interestingly, a gross post-mortem external examination of the *Wt1*^*LoxP/GFP*^*;Wt1*^*Cre*^ mice revealed the presence of structures that resembled a micropenis in both XY and XX mice (Fig 2G). The analysis of the internal reproductive tract in controls and in XY and XX *Wt1*^*LoxP/GFP*^*;Wt1*^*Cre*^ mice revealed that the mutant mice presented two small structures compatible with gonads at the lower pole of both kidneys, with the mutant mice exhibiting reproductive genital tracts containing both male and female structures (Fig 2H).

Altogether, these data indicate that *Wt1*^*LoxP/GFP*^*;Wt1*^*Cre*^ mice represent a new *Wt1KO* mouse model in which *Wt1* is deleted at the bipotential gonad stage.

### *Wt1*^*LoxP/GFP*^*;Wt1*^*Cre*^ mice represent a new mouse model of DSDs

**Fig 3:**
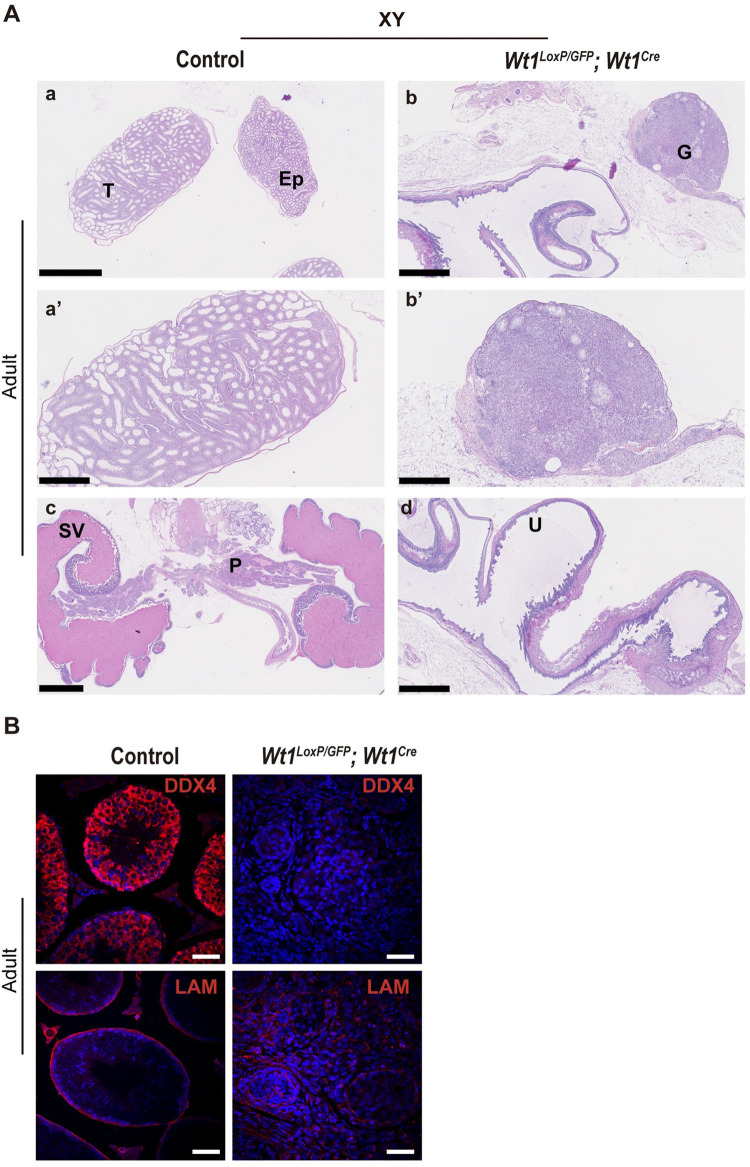
Testis dysgenesis and genital tracts containing both male and female structures in XY *Wt1*^*LoxP/GFP*^*;Wt1*^*Cre*^ mice. (A) Haematoxylin and eosin (H&E) staining of adult gonads and genital tract sections from XY control and *Wt1*^*LoxP/GFP*^*;Wt1*^*Cre*^ mice. Note the absence of mature gonads and the presence of both male and female genital tract structures in the mutant mice. G, gonad; Ep, epididymis; P, prostate; SV, seminal vesicles; T, testis; U, uterus. (B) Immunofluorescence staining for DDX4 and laminin (LAM) (red) and nuclear DAPI staining (blue), using sections of gonads from XY control and *Wt1*^*LoxP/GFP*^*;Wt1*^*Cre*^ adult mice. Representative images from a minimum of three each of XY control and mutant mice are shown. Scale bars: 2.5 mm in a, c; 1 mm in b, a’, d; 500 μm in b’ and 50 μm in B.

**Fig 4:**
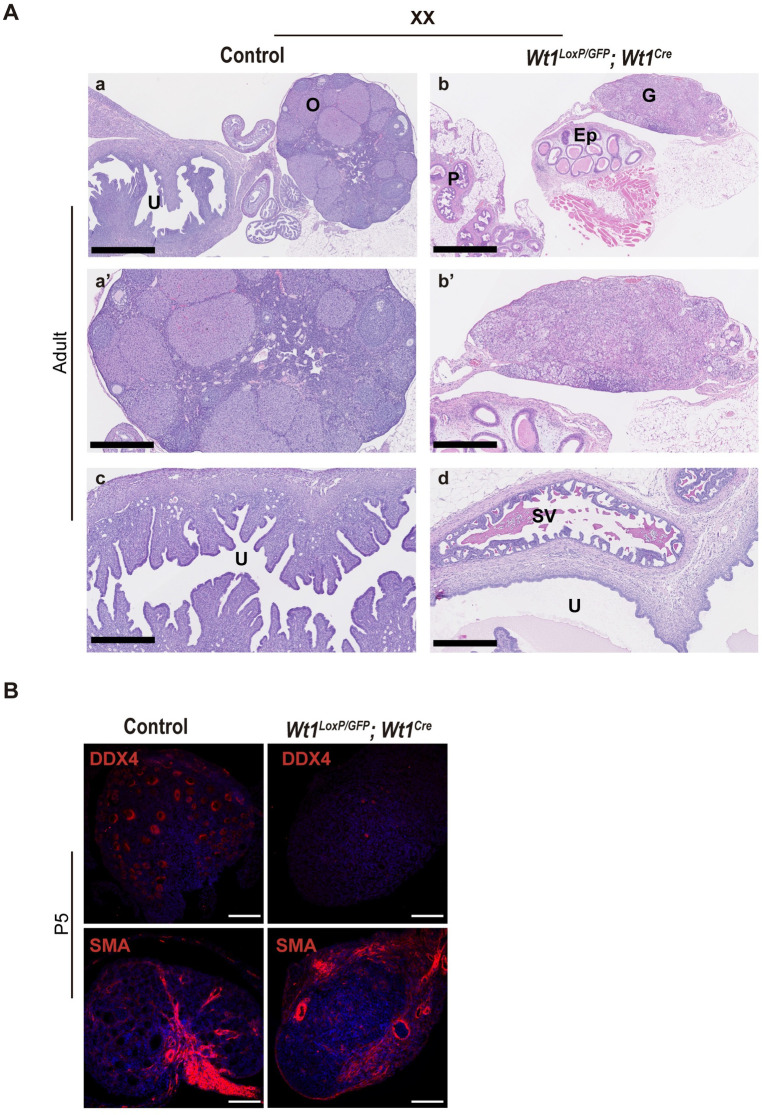
Ovarian dysgenesis and genital tracts containing both male and female structures in XX *Wt1*^*LoxP/GFP*^*;Wt1*^*Cre*^ mice. (A) H&E staining of adult gonads and genital tract sections from XX control and *Wt1*^*LoxP/GFP*^*;Wt1*^*Cre*^ mice. Note the absence of follicles and oocytes and the presence of both male and female genital tract structures in the mutant mice. G, gonad; Ep, epididymis; O, ovary; P, prostate; SV, seminal vesicles; U, uterus. (B) Immunofluorescence staining for DDX4 and SMA (red) and nuclear DAPI staining (blue), using sections of gonads from XX control and *Wt1*^*LoxP/GFP*^*;Wt1*^*Cre*^ P5 mice. A dramatic reduction in oocytes is observed in the gonads of *Wt1*^*LoxP/GFP*^*;Wt1*^*Cre*^ mice. Representative images from a minimum of three each of XX control and mutant mice are shown. Scale bars: 1 mm in a, b; 500 μm in a’-d and 100 μm in B.

Next, we performed a detailed histological analysis of the reproductive tract of the adult mutant mice. Interestingly, the analysis of XY and XX *Wt1*^*LoxP/GFP*^*;Wt1*^*Cre*^ mice revealed the absence of mature gonads when compared to control mice (Figs 3 and 4). While the testes of control mice exhibited a typical seminiferous tubule morphology with spermatogenic cells, these structures were absent in the gonads of XY *Wt1*^*LoxP/GFP*^*;Wt1*^*Cre*^ mice (Figs 3A and S4A). To better characterise the mutant gonad phenotype, immunostaining analysis was performed using a panel of different antibodies. The immunostaining of gonads from XY *Wt1*^*LoxP/GFP*^*;Wt1*^*Cre*^ mice for laminin validated the histological results. In the testis of control mice, laminin strongly localised at the basement membrane delineating the seminiferous tubule; however, in the gonads of XY *Wt1*^*LoxP/GFP*^*;Wt1*^*Cre*^ mice, the seminiferous tubule was absent and laminin exhibited an irregular distribution pattern (Fig 3B). The immunostaining for DDX4, corroborated the histology data, demonstrating that the defects in the formation of the seminiferous tubule also correlated with an absence of spermatogonia cells in the gonads of XY *Wt1*^*LoxP/GFP*^*;Wt1*^*Cre*^ mice (Figs 3B and S4A).

The histological examination of adult XX mutant gonads also demonstrated severe ovarian dysgenesis in the XX *Wt1*^*LoxP/GFP*^*;Wt1*^*Cre*^ mice (Figs 4A and S4B). Follicles and oocytes were evident in the ovaries of adult control mice, but were absent in the mutant XX gonads (Figs 4A and S4B). Ovaries produce an abundant number of follicles with immature oocytes in the first postnatal week. In line with the data from the histological examination of adult mice, the immunostaining of mutant gonads with DDX4 at postnatal (P5) revealed a dramatic reduction of DDX4 positive oocytes in mutant mice (Fig 4B). In addition, while each follicle in the ovaries of control mice was clearly delineated by Alpha Smooth Muscle Actin (SMA) immunostaining, the absence of follicles and an atypical SMA staining was clearly evident in the gonads of the mutant mice (Fig 4B).

The histological analysis also revealed that the remaining genital tract in all the XX and XY *Wt1*^*LoxP/GFP*^*;Wt1*^*Cre*^ mice consisted of the simultaneous presence of both male and female genital structures. Both male and female organs, including the oviducts, uterine horns, epididymides, vas deferens, seminal vesicles (SVs) and the prostate, were identified histologically in the XY and XX *Wt1*^*LoxP/GFP*^*;Wt1*^*Cre*^ mice (Figs 3A, 4A and S5).

Altogether, these data indicate that the *Wt1*^*LoxP/GFP*^*;Wt1*^*Cre*^ mice represent a new mouse model of DSDs that reaches adulthood. Both the XX and XY mutants displayed ambiguous external genitalia, which correlated with defects in the development of mature gonads and the presence of both male and female internal genital tract structures.

### Male and female early gonadal development is disrupted in *Wt1*^*LoxP/GFP*^*;Wt1*^*Cre*^ mice

**Fig 5:**
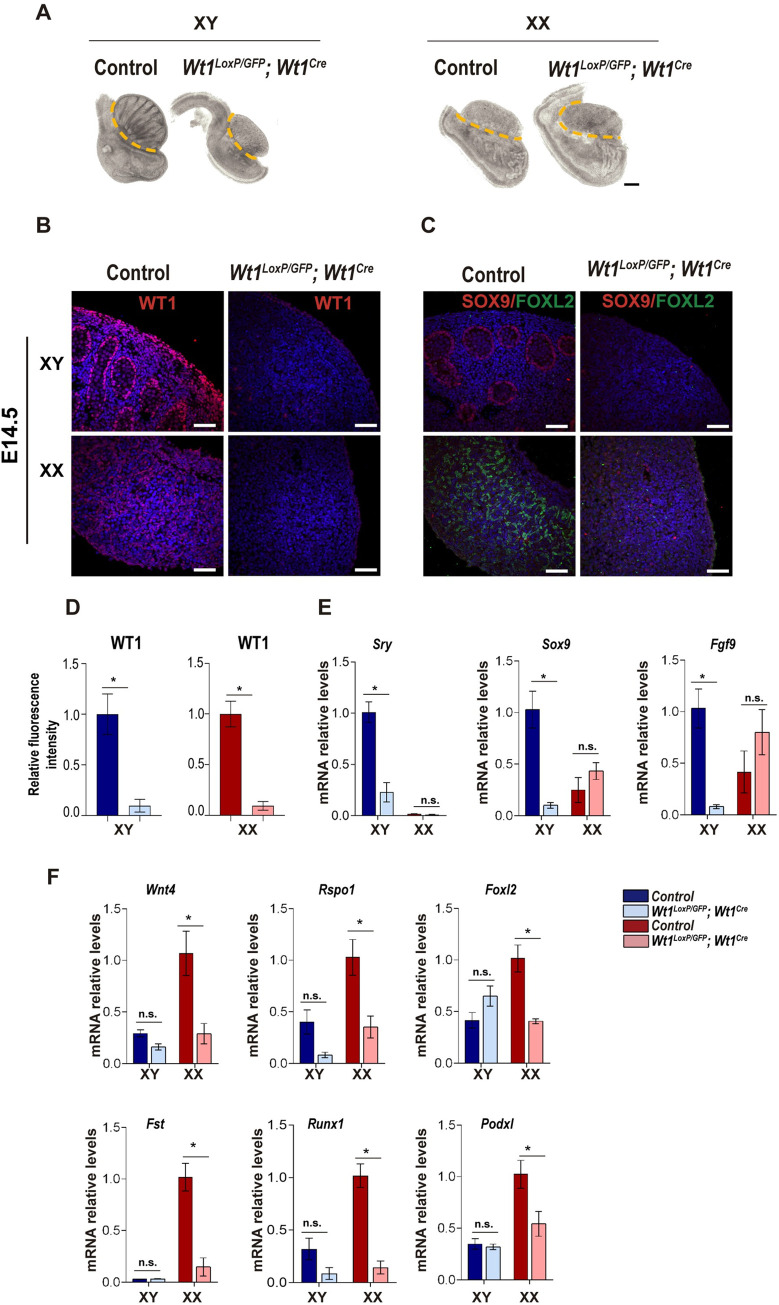
Embryonic gonad differentiation is impaired in *Wt1*^*LoxP/GFP*^*;Wt1*^*Cre*^ mice. (A) Bright-field images of gonads from XY and XX control and *Wt1*^*LoxP/GFP*^*;Wt1*^*Cre*^ E14.5 mice. Dashed lines denote the border between the gonads and the mesonephros. Testis cords are absent in the gonads of XY *Wt1*^*LoxP/GFP*^*;Wt1*^*Cre*^ mice. (B) Immunofluorescence staining for WT1 (red) and nuclear DAPI staining (blue), using sections from gonads of XY and XX control and *Wt1*^*LoxP/GFP*^*;Wt1*^*Cre*^ E14.5 mice. (C) Immunostaining for the Sertoli cell marker SOX9 (red) and the pre-granulosa cell marker FOXL2 (green) and nuclear DAPI staining (blue), using sections of gonads from XY and XX control and *Wt1*^*LoxP/GFP*^*;Wt1*^*Cre*^ E14.5 mice. No SOX9- or FOXL2-positive cells are formed in the *Wt1*^*LoxP/GFP*^*;Wt1*^*Cre*^ XY and XX gonads, respectively. (D) Quantification of the WT1 fluorescence intensity in gonads from control and *Wt1*^*LoxP/GFP*^*;Wt1*^*Cre*^ E14.5 mice. Values represent the mean ± s.e.m. (n = 3). *P < 0.05, Student’s *t*-test. qRT-PCR analysis of the key (E) testis and (F) ovarian genes in the XY and XX gonads at E12.5 from control and *Wt1*^*LoxP/GFP*^*;Wt1*^*Cre*^ mice. In the gonads of XY *Wt1*^*LoxP/GFP*^*;Wt1*^*Cre*^ mice, there is a significant reduction in the levels of *Sry*, *Sox9* and *Fgf9* expression. Gonads from XX *Wt1*^*LoxP/GFP*^*;Wt1*^*Cre*^ mice display a dramatic downregulation of *Wnt4*, *Rspo1*, *Foxl2*, *Fst*, *Runx1* and *Podxl* expression. Values represent the mean ± s.e.m. *P < 0.05, two-way ANOVA followed by Tukey’s post-hoc test. n = 3–4. Representative immunostaining images from a minimum of three each of XX and XY control and mutant mice are shown. Scale bars: 250 μm in A; and 50 μm in B, C.

Differentiation of the bipotential gonad represents a critical step in the formation of the testes and ovaries [3]. After observing the dramatic DSD features in *Wt1*^*LoxP/GFP*^*;Wt1*^*Cre*^ mice, we evaluated whether the phenotype observed in adult mice reflected a defect in gonadal morphogenesis. Compared to control mice, the gross morphological examination of the gonads of *Wt1*^*LoxP/GFP*^*;Wt1*^*Cre*^ mice at E14.5 revealed that the XY mutant gonads were morphologically different and had an appearance similar to that of a female gonad. However, no significant differences were observed in the XX mutant gonads (Fig 5A). A disrupted testis cord structure was already evident early on during the morphological analysis of XY *Wt1*^*LoxP/GFP*^*;Wt1*^*Cre*^ mice, which was confirmed by laminin immunostaining (S6 Fig). Further examination of the mutant gonads at E14.5 with the anti-WT1 antibody confirmed the reduced protein expression of WT1 observed in the gonads of *Wt1*^*LoxP/GFP*^*;Wt1*^*Cre*^ mice at E11.5 (Fig 5B and 5D). Immunostaining for SOX9 confirmed a defect in the testis cord formation and an absence of SOX9-positive Sertoli cells in the gonads of XY *Wt1*^*LoxP/GFP*^*;Wt1*^*Cre*^ mice (Fig 5C). Interestingly, FOXL2 immunostaining revealed the absence of FOXL2-positive pre-granulosa cells in the gonads of XX *Wt1*^*LoxP/GFP*^*;Wt1*^*Cre*^ mice (Fig 5C).

Next, we analysed whether the defects in gonad differentiation correlated with changes in the gene expression patterns of each specific programme. The expression of genes that play a relevant role in the initiation of the testis and ovarian developmental programmes was analysed by qRT-PCR at E12.5 (Fig 5E and 5F). Interestingly, the analysis of Sertoli cell markers revealed that *Sox9* mRNA expression at E12.5 was almost undetectable in the gonads of XY *Wt1*^*LoxP/GFP*^*;Wt1*^*Cre*^ mice, which was consistent with the absence of SOX9-positive cells in the mutant gonads at E14.5 (Fig 5C and 5E). The lack of *Sox9* expression in the gonads of XY *Wt1*^*LoxP/GFP*^*;Wt1*^*Cre*^ mice correlated with the downregulation of other Sertoli cell markers such as *Sry* and the SOX9 downstream target *Fgf9* (Fig 5E).

The expression of ovary-specific genes like *Wnt4*, *Rspo1*, *Fst*, *Foxl2*, *Runx1 and Podxl* was significantly downregulated in the gonads of XX *Wt1*^*LoxP/GFP*^*;Wt1*^*Cre*^ mice compared to control mice, which correlated with the absence of FOXL2-positive cells in the mutant XX mice (Fig 5C and 5F). The initiation of either the testis- or ovary-determining pathway requires both the upregulation of their specific pathway and the repression of the other pathway [25]. Our analysis demonstrated that both the XY and XX *Wt1*^*LoxP/GFP*^*;Wt1*^*Cre*^ gonads failed to upregulate their specific sex-determining genetic programme.

### Steroidogenic genes are differently regulated in XY and XX *Wt1*^*LoxP/GFP*^*;Wt1*^*Cre*^ gonads

**Fig 6:**
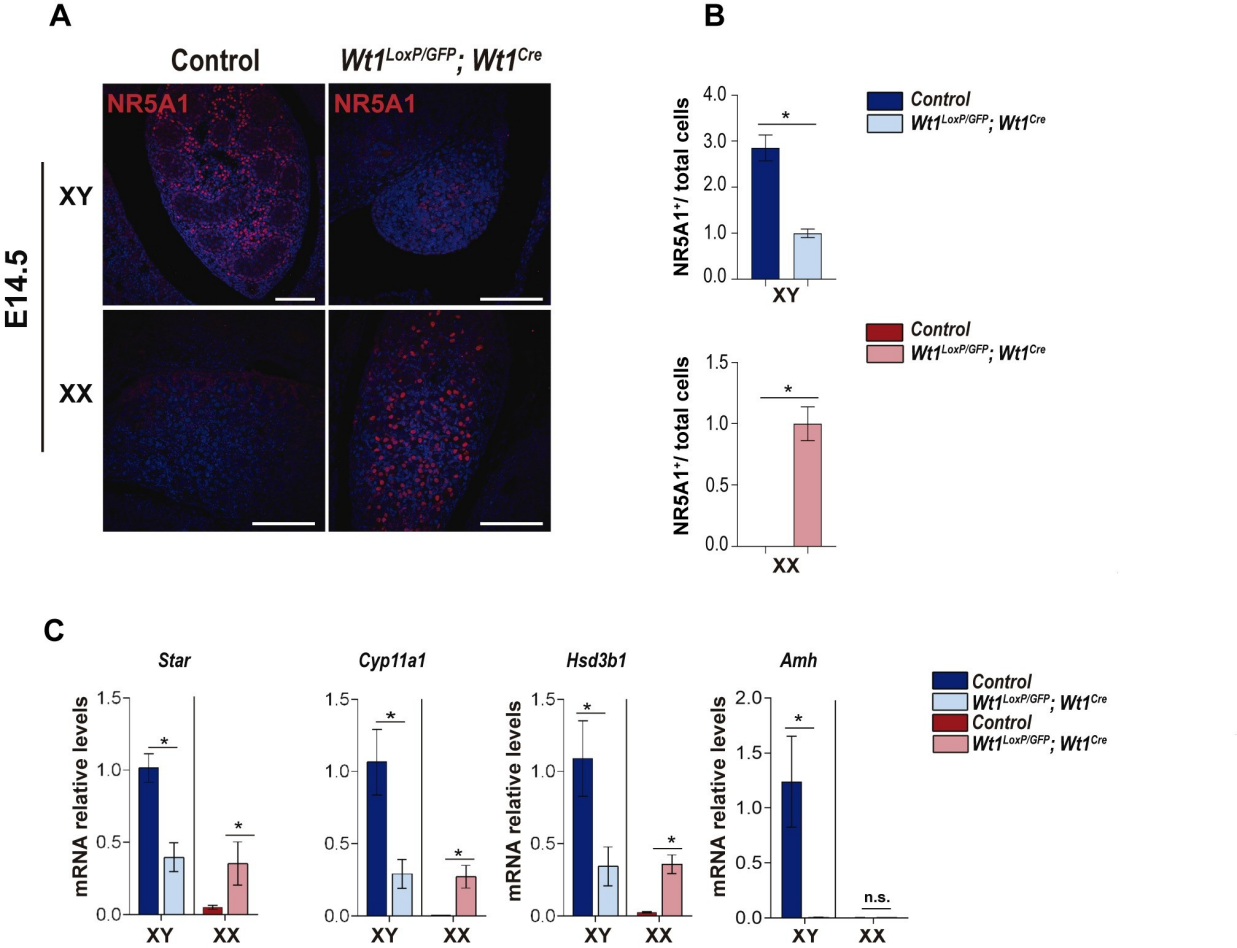
Steroidogenic programme is differently regulated in the XX and XY gonads of *Wt1*^*LoxP/GFP*^*;Wt1*^*Cre*^ mice. (A) Immunostaining for NR5A1 (red) and nuclear DAPI staining (blue), using sections of gonads from XY and XX control and *Wt1*^*LoxP/GFP*^*;Wt1*^*Cre*^ E14.5 mice. (B) Relative ratio of NR5A-positive cells/ total cells in gonads from control and *Wt1*^*LoxP/GFP*^*;Wt1*^*Cre*^ E14.5 mice. Values represent the mean ± s.e.m. (XY n = 4 and XX n = 3). *P < 0.05, Student’s *t*-test. (C) qRT-PCR analysis of steroidogenic and anti-Müllerian hormone genes in XY and XX gonads at E14.5 from control and *Wt1*^*LoxP/GFP*^*;Wt1*^*Cre*^ mice. The mRNA levels of steroidogenic genes, such as *Star*, *Cyp11a1*, *Hsd3b1* and the anti-Müllerian hormone gene (*Amh)* are significantly decreased in the XY gonads of *Wt1*^*LoxP/GFP*^*;Wt1*^*Cre*^ mice. In the gonads of XX *Wt1*^*LoxP/GFP*^*;Wt1*^*Cre*^ mice, there is a significant upregulation of *Star*, *Cyp11a1* and *Hsd3b1* expression. Values represent the mean ± s.e.m. (n = 3–4). *P < 0.05, Student’s *t*-test. Representative immunostaining images from a minimum of three each of XX and XY control and mutant mice are shown. Scale bars: 100 μm in A.

The ambiguous external genitalia and the presence of reproductive genital tracts containing both male and female structures observed in the XY and XX *Wt1*^*LoxP/GFP*^*;Wt1*^*Cre*^ mice prompted us to analyse the expression of the steroidogenic factor 1 (NR5A1/SF1) in the control and mutant mice. Immunostaining analysis of NR5A1 at E14.5 confirmed previous findings, NR5A1 was abundantly expressed in control testes in comparison with ovaries (Fig 6A) [26]. In addition, we observed a significant decrease in the number of NR5A1-positive cells in the XY *Wt1*^*LoxP/GFP*^*;Wt1*^*Cre*^ gonads. However, we found the opposite findings and we observed the presence of numerous cells that were strongly positive for NR5A1 in XX *Wt1*^*LoxP/GFP*^*;Wt1*^*Cre*^ gonad (Fig 6A and 6B).

NR5A1 is a nuclear hormone receptor that controls the expression of the steroidogenic enzymes and cholesterol transporters required for steroidogenesis [27]. Thus, we performed qRT-PCR analysis of several genes involved in different steps of steroid production such as *Star*, *Cyp11a1 and Hsd3b1*. Similar to NR5A1, the expression of these genes was downregulated in the gonads of XY *Wt1*^*LoxP/GFP*^*;Wt1*^*Cre*^ mice (Fig 6A–6C). Interestingly, *Star*, *Cyp11a1* and *Hsd3b1* expression was upregulated in the gonads of XX *Wt1*^*LoxP/GFP*^*;Wt1*^*Cre*^ mice which correlated also with the increase in NR5A1 observed in mutant XX gonads (Fig 6A–6C).

Müllerian duct regression is induced by the expression of *Amh*, encoding anti-Müllerian hormone, from the embryonic Sertoli cells [28]. Given the retention of structures derived from the Müllerian ducts in XY *Wt1*^*LoxP / GFP*^*; Wt1*^*Cre*^ mice, we also analysed *Amh* expression in control and mutant gonads at E14.5. As expected, we observed a dramatic reduction in *Amh* levels in XY *Wt1*^*LoxP / GFP*^*; Wt1*^*Cre*^ mice (Fig 6C).

Altogether, these findings demonstrate that *Wt1* deletion in the bipotential gonads impairs the typical steroidogenic programme of XY and XX gonads. Our data also indicate that the AMH signalling is disrupted in XY mutant mice.

### Deletion of *Wt1* during early gonadogenesis leads to a reduction of germ cells in *Wt1*^*LoxP/GFP*^*;Wt1*^*Cre*^ mice

**Fig 7:**
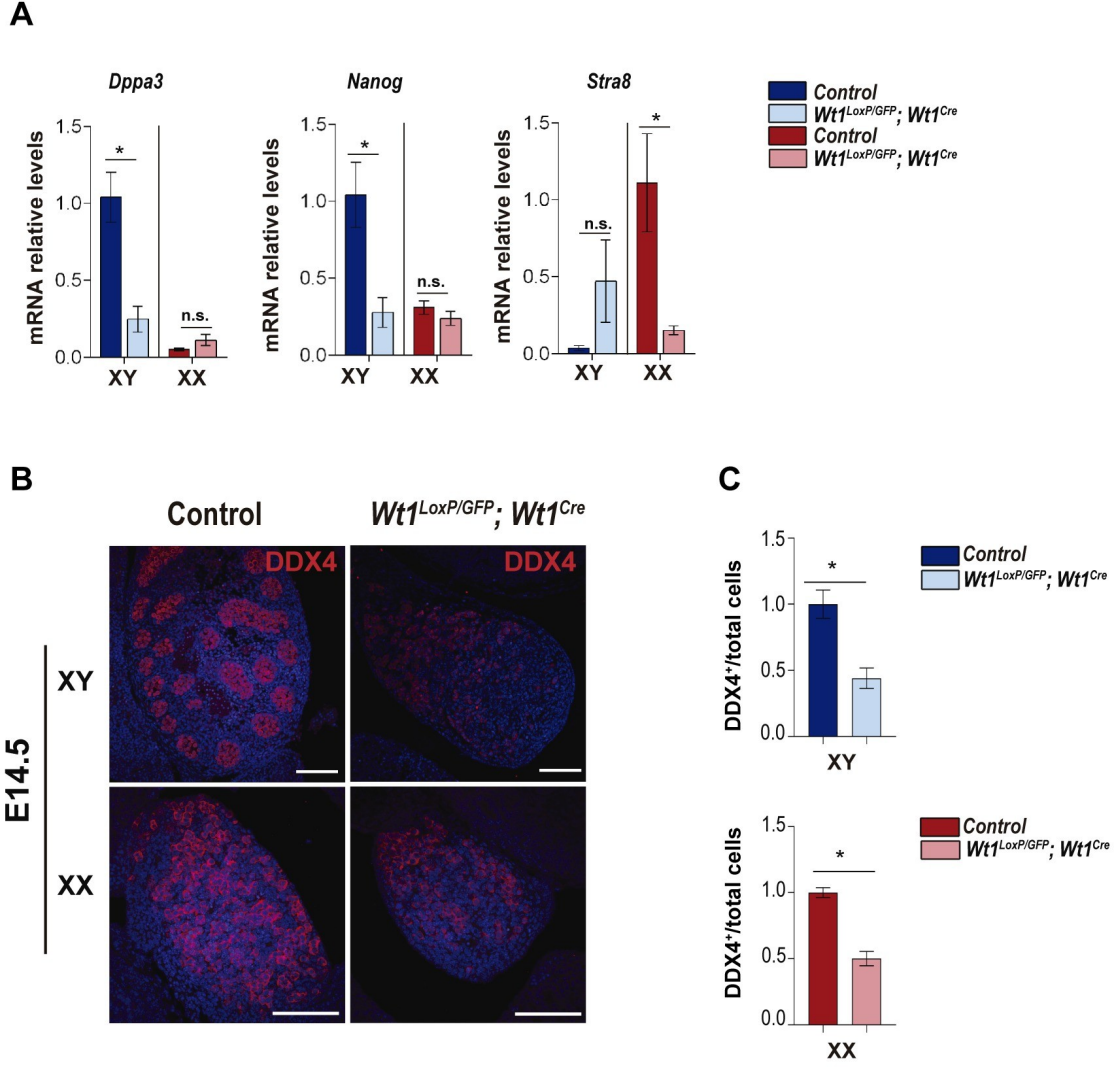
Embryonic *Wt1*^*LoxP/GFP*^*;Wt1*^*Cre*^ mice exhibit germ cell reduction. (A) qRT-PCR analysis of XY and XX germ cell markers in the gonads of control and *Wt1*^*LoxP/GFP*^*;Wt1*^*Cre*^ E14.5 mice. Note the significant downregulation of the pluripotent cell markers *Dppa3* and *Nanog* in the XY *Wt1*^*LoxP/GFP*^*;Wt1*^*Cre*^ gonads and the meiotic marker *Stra8* in the XX mutants. Values represent the mean ± s.e.m. (n = 3–4). *P < 0.05, Student’s *t*-test. (B) Immunofluorescence analysis for DDX4 (red) and nuclear DAPI staining (blue), using sections of gonads from XY and XX control and *Wt1*^*LoxP/GFP*^*;Wt1*^*Cre*^ E14.5 mice. (C) Relative ratio of DDX4 positive cells/ total cells in gonads from control and *Wt1*^*LoxP/GFP*^*;Wt1*^*Cre*^ E14.5 mice. Values represent the mean ± s.e.m. (n = 3). *P < 0.05, Student’s *t*-test. Representative immunostaining images from a minimum of three each of XX and XY control and mutant mice are shown. Scale bars: 100 μm in B.

To identify the causes of the absence of oocytes and spermatogenic cells in *Wt1*^*LoxP/GFP*^*;Wt1*^*Cre*^ mice, we analysed germ cell production in the embryonic gonads. Germ cells in the foetal testes do not enter meiosis and are mitotically arrested, maintaining the expression of pluripotency genes. By contrast, germ cells in the ovaries enter meiosis and express meiotic markers [3]. qRT-PCR analysis of the pluripotency genes *Dppa3* and *Nanog* at E14.5 demonstrated a decreased expression in the gonads of XY *Wt1*^*LoxP/GFP*^*;Wt1*^*Cre*^ mice compared to control mice (Fig 7A). Additionally, expression of the meiotic marker *Stra8* was downregulated in the gonads of XX *Wt1*^*LoxP/GFP*^*;Wt1*^*Cre*^ mice (Fig 7A). Next, we stained gonads at E14.5 with an antibody against the germ cell marker DDX4. In XY control gonads, DDX4 positive cells were enclosed within the testis cord while in XX gonads, DDX4 positive cells were dispersed within the gonads (Fig 7B). Both XX and XY mutant gonads had reductions in the numbers of DDX4-positive germ cells compared to control mice (Fig 7B and 7C).

Overall, these results indicate that germ cell number is reduced from the embryonic stages in the gonads of XY and XX *Wt1*^*LoxP/GFP*^*;Wt1*^*Cre*^ mice.

### Abnormal differentiation of postnatal ovaries in *Wt1*^*LoxP/GFP*^*;Wt1*^*Cre*^ mice

**Fig 8:**
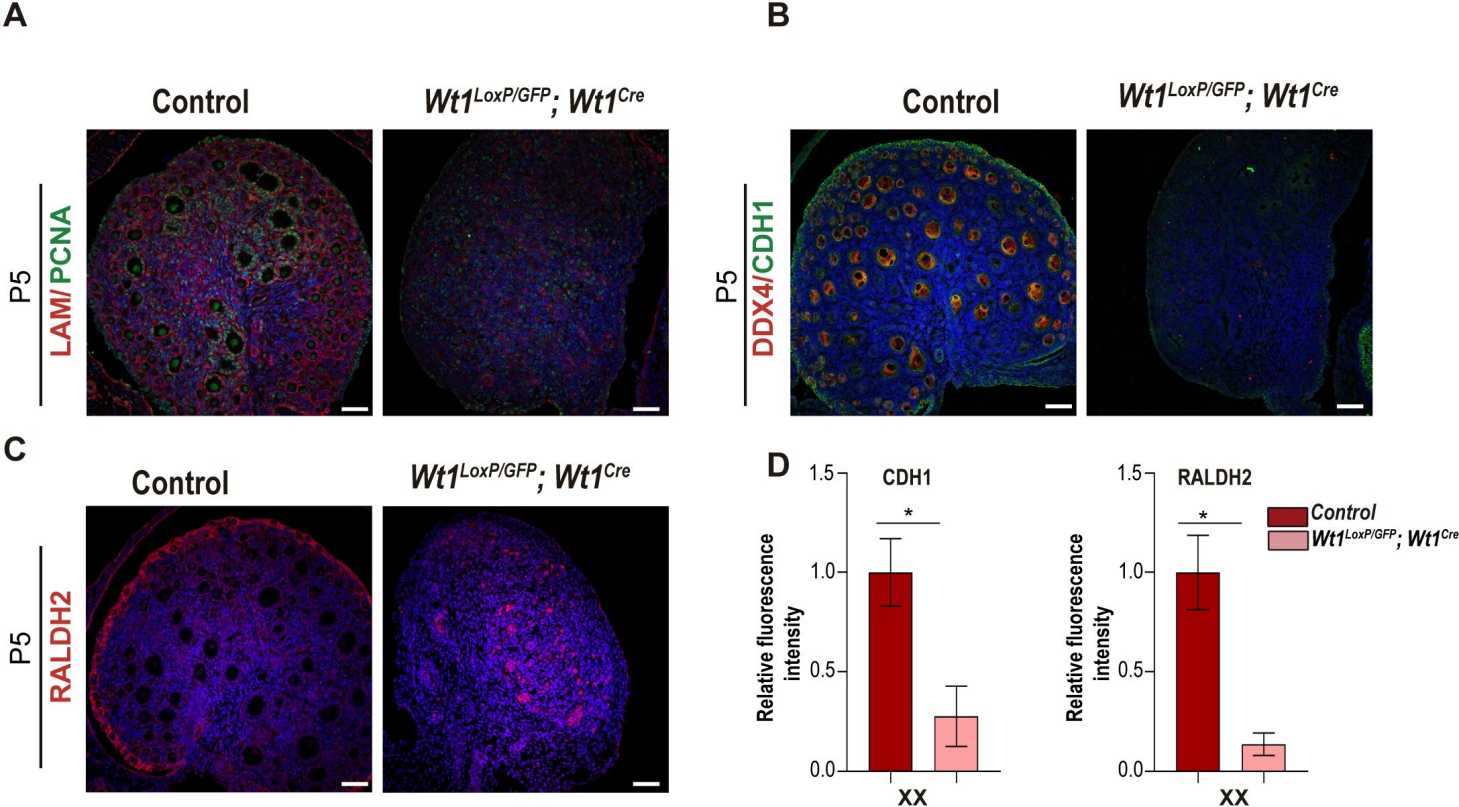
Deletion of *Wt1* during early gonadogenesis has a great impact on ovarian morphogenesis. Immunofluorescence staining for laminin (LAM) (red) and proliferating cell nuclear antigen (PCNA) (green) (A), E-cadherin (CDH1) (green) and DDX4 (red) (B), and RALDH2 (red) and nuclear DAPI staining (blue) (C), using sections of gonads from XX control and *Wt1*^*LoxP/GFP*^*;Wt1*^*Cre*^ P5 mice. Only control mice showed ovaries with well-differentiated cortical and medullar regions and an abundant number of primary follicles and oocytes. (D) Quantification of the CDH1 and RALDH2 fluorescence intensity in the ovarian epithelium (OE) from control and *Wt1*^*LoxP/GFP*^*;Wt1*^*Cre*^ mice. Values represent the mean ± s.e.m. (n = 3). *P < 0.05, Student’s *t*-test. Decreased CDH1 and RALDH2 expression was observed in the OE of *Wt1*^*LoxP/GFP*^*;Wt1*^*Cre*^ P5 mice. An increased number of RALDH2-positive somatic cells was observed in *Wt1*^*LoxP/GFP*^*;Wt1*^*Cre*^ P5 mice. Representative immunostaining images from a minimum of three of each XX and XY control and mutant mice are shown. Scale bars: 50 μm.

In contrast to male development, very little is known about the role of WT1 in female gonad development [5]. To gain more insight into the XX mutant gonad phenotype, we decided to first interrogate the nature of the NR5A1-positive cells observed in XX mice at E14.5. Double IF analysis with the antibodies against NR5A1 and podocalyxin (PODXL) demonstrated that NR5A1-positive cells found in mutant mice were mainly restricted to cells expressing abundant levels of PODXL. Interestingly, while NR5A1-positive fetal Leydig cells do not express PODXL, progenitor cells of the early gonad expressed high levels of NR5A1 and PODXL suggesting that NR5A1-positive cells of the mutant gonads resemble early gonad progenitor cells (S7 Fig) [29]. Next, we analysed the gonads of XX Wt1^*LoxP/GFP*^*;Wt1*^*Cre*^ mice at P5, when the ovaries are more developed and primordial follicles are largely complete. Double immunostaining with antibodies against laminin and proliferating cell nuclear antigen (PCNA) demarcated the medulla and the cortical regions of the P5 control ovaries. It also demonstrated the aberrant morphology of the ovaries in Wt1^*LoxP/GFP*^*;Wt1*^*Cre*^ mice (Fig 8A). The abundant number of primary follicles and oocytes observed in the control mice were totally absent in the mutant mice (Fig 8A). To further characterise the impact of *Wt1* deletion on ovarian differentiation we stained P5 ovaries with antibodies against E-cadherin (CDH1), DDX4 and RALDH2 (Fig 8B and 8C). Immunostaining of CDH1 and RALDH2 revealed that the abnormal morphology of mutant ovaries correlated with a dramatic downregulation of CDH1 and RALDH2 expression (Fig 8B–8D). Interestingly, the decreased expression levels of RALDH2 observed in the ovarian epithelium (OE) of the mutant gonads correlated with the presence of clusters of somatic cells expressing abundant levels of RALDH2 in the mutant gonads, which probably represent undifferentiated progenitor cells [30].

Altogether, these data suggest that deletion of WT1 in the early progenitor cells of XX gonads dramatically disrupts ovarian development.

## Discussion

*WT1* mutations have been reported in several human conditions in which patients present a variable range of genital malformations varying from ambiguous external genitalia to gonadal dysgenesis. In this study, we generated and characterised a new *Wt1KO* mouse model in which *Wt1* deletion was accomplished at the early bipotential gonad stage (*Wt1*^*LoxP/GFP*^*;Wt1*^*Cre*^). The analyses of these mice demonstrated the importance of *Wt1* for early gonad differentiation and the impact of this early deletion on the formation of the adult reproductive system. Adult *Wt1*^*LoxP/GFP*^*;Wt1*^*Cre*^ mice lacked mature gonads, with both XX and XY mutants displaying genital tracts containing both male and female structures, as well as ambiguous external genitalia.

Despite the impact of *WT1* mutations on sex development, many questions about the loss-of-function effect of this gene on early bipotential gonad differentiation and its impact on adult sex development remain unanswered. The initial characterisation of the germline *Wt1KO* mouse model indicated a critical role for WT1 during early gonadogenesis [20]. However, assessing its functions at this early stage and its impact on adult sex development has been difficult due to the complete gonadal agenesis exhibited by the conventional *Wt1KO* mice and the embryonic lethality observed in *Wt1KO* mouse models. In an attempt to examine the role of WT1 before sex determination, a ubiquitous tamoxifen-inducible Cre *Wt1KO* model was recently generated [24]. However, the early lethality of these mutants precluded the analysis of the impact of *Wt1* deletion at an early stage on more mature gonad formation and adult sex development. In this study, we used a *Wt1*^*Cre*^ BAC transgenic mouse line that dramatically reduced *Wt1* levels in the early bipotential gonads of *Wt1*^*LoxP/GFP*^*;Wt1*^*Cre*^ mice. Our mouse model is the first with a *Wt1KO* in the bipotential gonads in which it is possible to follow the behaviour of the mutant gonads *in vivo* throughout different developmental phases, circumventing the need for organ cultures, which were required in the case of the tamoxifen-inducible *Wt1KO* mouse model [24]. In this mouse model, as well as in humans with WT1 loss-of-function mutations, the role of WT1 is compromised during the entire lifespan of the individual. In *Wt1*^*LoxP/GFP*^*;Wt1*^*Cre*^ mice, *Wt1* downregulation was achieved at the same developmental stage in the XX and XY primitive gonads, enabling the analysis of the effects of WT1 deletion on primitive progenitor cells on sex development.

The *Wt1*^*Cre*^ transgenic line used in this study was generated more than ten years ago and has been used by several groups since then [16,21,31,32]. We found that *Wt1*^*Cre*^ efficiently recombines in the embryonic gonads, but, unexpectedly, we observed almost no recombination in the embryonic kidney and adrenal gland. Descendants of WT1-positive cells contribute to the development of the gonads, adrenal gland and kidneys in a temporally distinct manner [6]. Despite the crucial role of WT1 in the formation of several organs, little is known about the molecular mechanisms underlying WT1 regulation. Such diversification in its temporal expression and functions probably reflects the presence of specific regulatory elements of the WT1 gene in the different populations of WT1-positive cells [6,33]. Our data and previous results from other groups suggest that this *Wt1*^*Cre*^ line is able to direct expression in the coelomic epithelium and its derivatives [34].

Many phenotypic features found in the XY *Wt1*^*LoxP/GFP*^*;Wt1*^*Cre*^ mice, such as gonadal dysgenesis and ambiguous genitalia, have been identified in XY humans with *WT1* mutations, including in those with Denys-Drash syndrome or Frasier syndrome. Male sex differentiation requires androgen activity that must occur within a critical timeframe during early embryo development [3]. A defect in the production of testosterone, due to a reduction in NR5A1 and the expression of several steroidogenic genes, could be one of the main causes of the features of DSDs observed in both our mutant mice and in XY humans with WT1 mutations. The embryonic gonads of XY *Wt1*^*LoxP/GFP*^*;Wt1*^*Cre*^ mice lacked the expression of early male-specific markers, which correlated with a defect in the production of Sertoli and foetal Leydig cells. Sertoli cells are the first somatic cell lineage to arise in the testes and are believed to play a crucial role in their subsequent differentiation and organisation [3]. Disruption in the formation of early Sertoli cells in *Wt1KO* gonads could trigger all of the DSD defects observed in the mutant XY mice. Interestingly, despite the gonads of XY *Wt1*^*LoxP/GFP*^*;Wt1*^*Cre*^ mice failing to upregulate the testis-specific programme, there was no upregulation of the ovary-determining pathway, with the mutant gonads remaining probably undifferentiated. Furthermore, the XY *Wt1*^*LoxP/GFP*^*;Wt1*^*Cre*^ mice showed reproductive genital tracts containing both male and female structures, which was probably due to an impairment in the regression of the Müllerian ducts. The reduction in *Amh* expression observed in the XY *Wt1*^*LoxP/GFP*^*;Wt1*^*Cre*^ mice could explain the presence of this phenotype.

The gonads of the XY *Wt1*^*LoxP/GFP*^*;Wt1*^*Cre*^ mice did not show testis cord formation, which correlated with an impaired production of Sertoli and a reduction in germ cells. Interestingly, we also observed a reduction in the expression of steroidogenic genes, which suggested an impaired production of foetal Leydig cells. Lineage tracing and single-cell transcriptomics of the mouse embryos have indicated that the supporting Sertoli cells and most steroidogenic Leydig cells of the developing testis are derived from common progenitor precursors originating from the coelomic epithelium [18,35]. Based on our results, we postulate that *Wt1* deletion in the XY primitive gonad leads to the progenitors of the somatic cells remaining immature.

There are fewer genes known to be associated with XX DSDs compared to XY DSDs, probably because genes and pathways leading to female sex development are poorly understood. Until very recently, patients carrying WT1 mutations and displaying DSDs were XY. However, during the last year, the number of studies reporting XX patients with *WT1* mutations has increased [9–11]. Our results indicate that *Wt1* deletion at the bipotential gonad stage can also impair female sex development. The XX *Wt1*^*LoxP/GFP*^*;Wt1*^*Cre*^ mice exhibited several characteristics associated with defects in female sex differentiation that are considered to be DSDs. The XX *Wt1*^*LoxP/GFP*^*;Wt1*^*Cre*^ mice in our study showed undifferentiated gonads, reproductive genital tracts containing both male and female structures and ambiguous external genitalia. Intriguingly, some but not all of these features have been recently described in XX humans with *WT1* mutations that are considered to be gain-of-function mutations but also loss-of-function mutations [9–11].

In recent years, single-cell transcriptomic analyses of the embryonic gonad have revealed new insights into female gonad development [29,36]. These data in combination with lineage tracing analysis have demonstrated the complexity of XX gonad differentiation as well as the presence of different populations of progenitor cells derived from the coelomic epithelium that are positive for WT1 [29]. The increased expression of NR5A1 and some steroidogenic genes observed in the gonads of XX *Wt1*^*LoxP/GFP*^*;Wt1*^*Cre*^ mice can contribute to the upregulation of testosterone production as well as the presence of genital tracts containing both male and female structures. Interestingly, the progenitor cells of embryonic gonads express several steroid hormone genes and their increased expression can masculinise female mice [29]. Based on our results, we speculate that the increased expression of the steroidogenic genes and NR5A1 observed in XX *Wt1*^*LoxP/GFP*^*;Wt1*^*Cre*^ mice could be due to impaired differentiation of the progenitor cells of the mutant gonads. In line with this observation, we also observed that the NR5A1-positive progenitor cells of early embryonic gonads expressed abundant levels of NR5A1 and PODXL [29].

In XX *Wt1*^*LoxP/GFP*^*;Wt1*^*Cre*^ mice, the OE showed abnormal differentiation as the mutant cells were positive for SMA and negative for CDH1. We also observed that RALDH2 was dramatically downregulated in the OE of mutant mice, indicating that the expression of this gene depends on WT1 activation, similar to that observed in the epicardium [37]. In contrast to the cells of the OE, some somatic cells of the mutant ovaries expressed abundant levels of RALDH2, suggesting that the regulation of this gene by WT1 in the gonads could be cell-dependent. Single-cell RNA sequencing of the mutant gonads is necessary to provide further insights into the stages in which the different populations of progenitor cells are arrested in *Wt1*^*LoxP/GFP*^*;Wt1*^*Cre*^ mice.

During embryonic development, WT1-expressing cells contribute to the formation of several tissues. Based on the findings presented here, we propose that a deeper characterisation of the *Wt1*^*LoxP/GFP*^*;Wt1*^*Cre*^ mouse model will aid in the understanding of the functions of WT1 in several populations of progenitor cells in different organs and tissues as well as the importance of these cell populations for adult organ formation.

## Materials and methods

### Ethics statement

All animal experiments were carried out in accordance with the regulations of the Animal Experimentation Ethics Committee (CEEA) of the University of Barcelona (ID C121CG2V6), thereby complying with current Spanish and European legislation.

### Animal models

The *Wt1*^*GFP/+*^, *Wt1*^*Cre*^ *(Tg(Wt1-cre)#Jbeb)*, *Wt1*^*LoxP/LoxP*^ and *R26*^*mTmG/mTmG*^ mice have been described previously [13,14,16,38,39] **S2 Table**. To generate male *Wt1*^*GFP/+*^*;Wt1*^*Cre*^ mice, *Wt1*^*Cre/Cre*^ males were mated with *Wt1*^*GFP/+*^ females. Mating between *Wt1*^*GFP/+*^*;Wt1*^*Cre*^ males and *Wt1*^*LoxP/LoxP*^ females generated a new *Wt1KO* mouse model (*Wt1*^*LoxP/GFP*^*;Wt1*^*Cre*^) and control animals. To generate *Wt1*^*Cre*^*;R26*^*mTmG/+*^ embryos, *Wt1*^*Cre/+*^ males were mated with *R26*^*mTmG/mTmG*^ females. Embryos were generated through timed matings, whereby females that had mated during the night were checked for plugs early the following morning. The morning on which a plug was found was considered to be E0.5. Genotyping was carried out using DNA extracted from the tail tip or ear biopsies. The primers used for genotyping and the determination of the presence of the Y chromosome are listed in **S3 Table**.

### Tissue preparation for histological analysis

Animal adult tissues (around 6 months old) were collected and fixed in 10% neutral-buffered formalin for approximately 24 hours, before being processed and embedded in paraffin. Embedded tissues were cut into 3- to 4-μm sections. Haematoxylin and eosin (H&E) staining was performed at the Histopathology Facility of the IRB, using the standard protocol. Images were acquired using an Olympus CX43 microscope. The histopathological analysis of the adult mice was performed by the Histology Services of the IRB.

### Immunofluorescence

Mouse embryos at the indicated stages and dissected postnatal gonads were fixed in 4% or 2% paraformaldehyde (PFA) respectively, embedded in paraffin, and cut into 7-μm sections. Following standard protocols of deparaffinisation, an antigen retrieval procedure was carried out by boiling the samples in a pressure cooker for 15 min in citrate buffer (10 mM tri-sodium citrate 2-hydrate; pH 6.0). The slides were then incubated in blocking serum (2% foetal bovine serum (FBS) in PBS) for 2 h before being incubated overnight with the primary antibody at 4°C. The slides were then washed with PBS, incubated at room temperature for 2 h with the appropriate secondary antibodies, and stained with DAPI (Thermo, ref. 62249) for 5 min for nuclear staining [40]. For a list of all the antibodies and dilutions used, see **S4 Table**. Samples were imaged using the Zeiss 880 confocal microscope.

ImageJ software was used for the quantification of Median Fluorescence Intensity (MFI) and the number of DDX4^-^positive (DDX4^+^) and NR5A1-positive (NR5A1^+^) cells. For each image, the number of DDX4^+^ or NR5A1^+^ cells versus total cells (DAPI) or the MFI was determined in a minimum of four regions of interest (ROIs). Two images per mouse were analysed. For the quantification of the MFI in the OE of P5 gonads, the OE surface was obtained by the manual drawing of the external contour of this epithelial cell layer. MFI intensity within the OE was quantified and normalized to the OE surface. A minimum of three control and mutant mice were analysed.

### FACS analysis

E14.5 embryonic kidneys from *Wt1*^*Cre*^*;R26*^*mTmG/+*^ and *Wt1*^*GFP/+*^ mice were digested in a collagenase solution (0.01%, dissolved in HBBS; Worthington, LS004196) for 30 min at 37°C using a shaking block at 1000 rpm (Eppendorf Thermomixer Compact). Collagenase activity was stopped by washing the cells in PBS containing 5% FBS. Cells were pelleted by centrifugation at 300 ***g*** for 5 min. Cells were filtered through a 40 μm cell strainer and subjected to FACS. FACS analysis was carried out by gating against negative control littermates. Doublets and dead cells were eliminated, using DAPI staining. The data were analysed using the BD FACSDiva software (v6.1.3).

### Isolation of RNA and real-time PCR

Whole gonads were used to isolate RNA using an RNA Isolation Kit (Invitrogen, 12183025). Purified RNA was used for reverse transcription and cDNA generation with SuperScript III Reverse Transcriptase (Thermo Fisher Scientific, 18080044). Relative expression levels were determined with the SYBR Select Master Mix (Thermo Fisher Scientific, 4472897). Real-time PCR experiments were performed in a LightCycler 480 System (Roche) [40]. The primers used are listed in **S5 Table**.

### Quantification and statistical analysis

Data are presented as the mean ± standard error of the mean. Statistical significance between two groups was determined by unpaired two-tailed Student’s *t*-test. We applied non-parametric two-way ANOVA followed by Tukey’s post-hoc test to evaluate the differences among multiple groups of samples.

## Supporting information

S1 Fig Monitoring *Wt1*^*Cre*^ activity in the embryonic urogenital region. (A) Immunofluorescence staining for GFP (green), WT1 (red) and nuclear DAPI staining (blue), using the urogenital region from *Wt1*^*Cre*^*;R26*^*mTmG/+*^ E14.5 mice. Cre activity (GFP-positive cells) is restricted to the gonads, the Müllerian ducts and some mesothelial cells covering the kidneys. AG, adrenal gland; G, gonad; K, kidney. (B) Gonads from *Wt1*^*Cre*^*;R26*^*mTmG/+*^ mice at E14.5 were co-labelled with antibodies against GFP (green) and the marker for germ cells DDX4 (red), as well as stained with the nuclear DAPI dye (blue). Representative immunostaining images from a minimum of three embryos are shown. Scale bars: 150 μm in A; and 50 μm in B.
(TIF)

S2 Fig *Wt1*^*Cre*^ transgene does not recombine in the kidneys. (A) Schematic representation of the transgenic constructs used to generate *Wt1*^*Cre*^ and *Wt1*^*GFP/+*^ mice. (B) FACS analysis of the digested kidneys from E14.5 *Wt1*^*Cre*^*;R26*^*mTmG/+*^ and *Wt1*^*GFP/+*^ mice. Plots from negative-control littermate kidneys are also shown. Note the absence of GFP-positive cells in the kidneys of *Wt1*^*Cre*^*;R26*^*mTmG/+*^ mice. (C) qRT-PCR analysis of *Wt1* in E14.5 kidneys from control and *Wt1*^*LoxP/GFP*^*;Wt1*^*Cre*^ mice. Values represent the mean ± s.e.m. (n = 4–5). *P < 0.05, Student’s *t*-test. (D) Immunofluorescence staining for WT1 (red) and nuclear DAPI staining (blue), using kidney sections from E14.5 control and *Wt1*^*LoxP/GFP*^*;Wt1*^*Cre*^ mice. Representative immunostaining images from a minimum of three control and mutant mice are shown. Scale bars: 100 μm.
(TIF)

S3 Fig *Wt1*^*Cre*^ is active during the earlier stages of gonad development. Immunofluorescence staining of GFP (green) and nuclear DAPI staining (blue), using sections from *Wt1*^*Cre*^*;R26*^*mTmG/+*^ E11.5 mice. GFP-positive cells were detected in the coelomic epithelium and somatic cells of the gonads. Representative immunostaining images from a minimum of three embryos are shown. Scale bar: 25μm.
(TIF)

S4 Fig Absence of seminiferous tubules and follicles in *Wt1*^*LoxP/GFP*^*;Wt1*^*Cre*^ mice. Higher magnification of the H&E staining of adult gonads from (A) XY and (B) XX control and *Wt1*^*LoxP/GFP*^*;Wt1*^*Cre*^ mice included in Figs 3A and 4A. Note the presence of seminiferous tubules and follicles in the control testis and ovary and their absence in the gonads of *Wt1*^*LoxP/GFP*^*;Wt1*^*Cre*^ mice. Images are representative of at least three each of XY and XX control and *Wt1*^*LoxP/GFP*^*;Wt1*^*Cre*^ adult mice. Scale bars: 100 μm.
(TIF)

S5 Fig The epididymis and seminal vesicles are also found in XY *Wt1*^*LoxP/GFP*^*;Wt1*^*Cre*^ mice. H&E staining of adult genital tract structures from XY control and *Wt1*^*LoxP/GFP*^*;Wt1*^*Cre*^ mice. (A, C) Magnified images of Fig 3A (a, c). Images are representative of three of each control and *Wt1*^*LoxP/GFP*^*;Wt1*^*Cre*^ adult mice. Scale bars: 500 μm in A,D; 250 μm in B and 1mm in C.
(TIF)

S6 Fig Abnormal basal lamina formation in the embryonic testis of *Wt1*^*LoxP/GFP*^*;Wt1*^*Cre*^ mice. Immunofluorescence staining for laminin (LAM) (red) and nuclear DAPI staining (blue), using gonad sections from E14.5 XY control and *Wt1*^*LoxP/GFP*^*;Wt1*^*Cre*^ mice. Representative immunostaining images from a minimum of three each of control and mutant mice are shown. Scale bars: 50 μm.
(TIF)

S7 Fig NR5A1-positive cells of the mutant gonads resemble early gonad progenitor cells. (A) Immunofluorescence staining for NR5A1 (red), podocalyxin (PODXL) (green) and nuclear DAPI staining (blue), using gonad sections from XY and XX control and XX *Wt1*^*LoxP/GFP*^*;Wt1*^*Cre*^ E14.5 mice. (a’-c’) Magnified images of the corresponding boxed areas of the lower magnification. (B) Immunofluorescence staining for NR5A1 (red), PODXL (green) and nuclear DAPI staining (blue) and WT1 (red) and nuclear DAPI staining (blue), using gonad sections from XX control mice at E11.5. Note that the NR5A1-positive cells of the mutant gonads co-express PODXL similar to the XX early progenitor cells, while foetal Leydig NR5A1-positive cells are negative for PODXL. Representative immunostaining images from a minimum of three of each control and mutant mice are shown. Scale bars: 50 μm in a-c, 10 μm in a’-c’ and 25 μm in B.
(TIF)

S1 Table Submendelian distribution of *Wt1*^*LoxP/GFP*^*;Wt1*^*Cre*^ mutant mice. Distribution of the different genetic combinations resulting from the intercross of *Wt1*^*GFP/+*^*;Wt1*^*Cre*^ males with *Wt1*^*LoxP/ LoxP*^ females.
(DOCX)

S2 Table Information of mouse models used in this article. (DOCX)

S3 Table List of primers used for genotyping. (DOCX)

S4 Table List of antibodies used in this study. (DOCX)

S5 Table List of primers used in the quantitative real-time PCR analysis. (DOCX)

S1 Data Numerical data underlying the results. (XLSX)
