# Supplementary figures and images for: Deletion of Wt1 during early gonadogenesis leads to differences of sex development in male and female adult mice
Source: PLoS Genet. 2022 Jun 15;18(6):e1010240. doi: 10.1371/journal.pgen.1010240 (PMC9200307; doi:10.1371/journal.pgen.1010240)

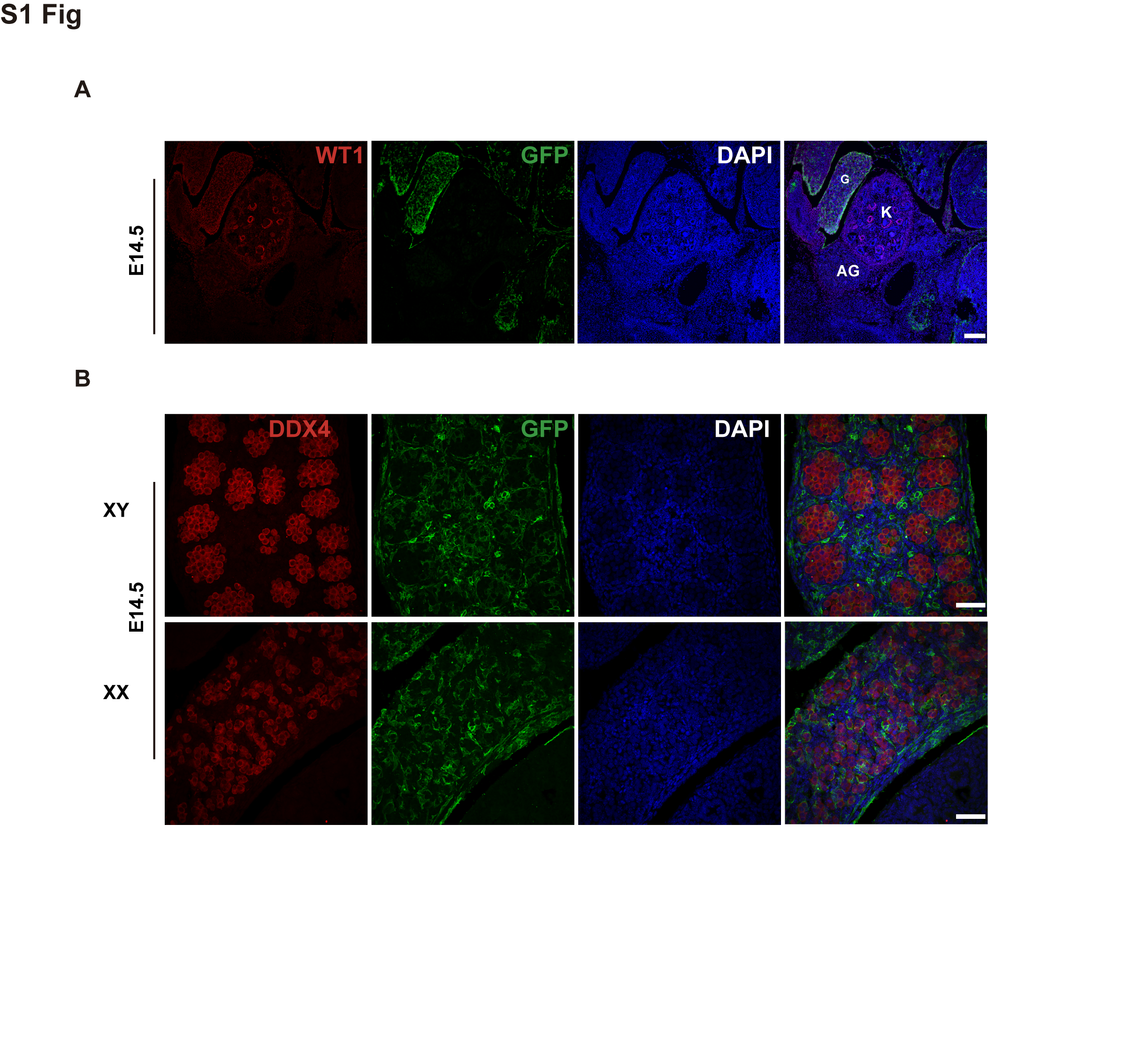

Supplement: S1 Fig — (A) Immunofluorescence staining for GFP (green), WT1 (red) and nuclear DAPI staining (blue), using the urogenital region from Wt1Cre;R26mTmG/+ E14.5 mice. Cre activity (GFP-positive cells) is restricted to the gonads, the Müllerian ducts and some mesothelial cells covering the kidneys. AG, adrenal gland; G, gonad; K, kidney. (B) Gonads from Wt1Cre;R26mTmG/+ mice at E14.5 were co-labelled with antibodies against GFP (green) and the marker for germ cells DDX4 (red), as well as stained with the nuclear DAPI dye (blue). Representative immunostaining images from a minimum of three embryos are shown. Scale bars: 150 μm in A; and 50 μm in B. (TIF) [file pgen.1010240.s001.tif]

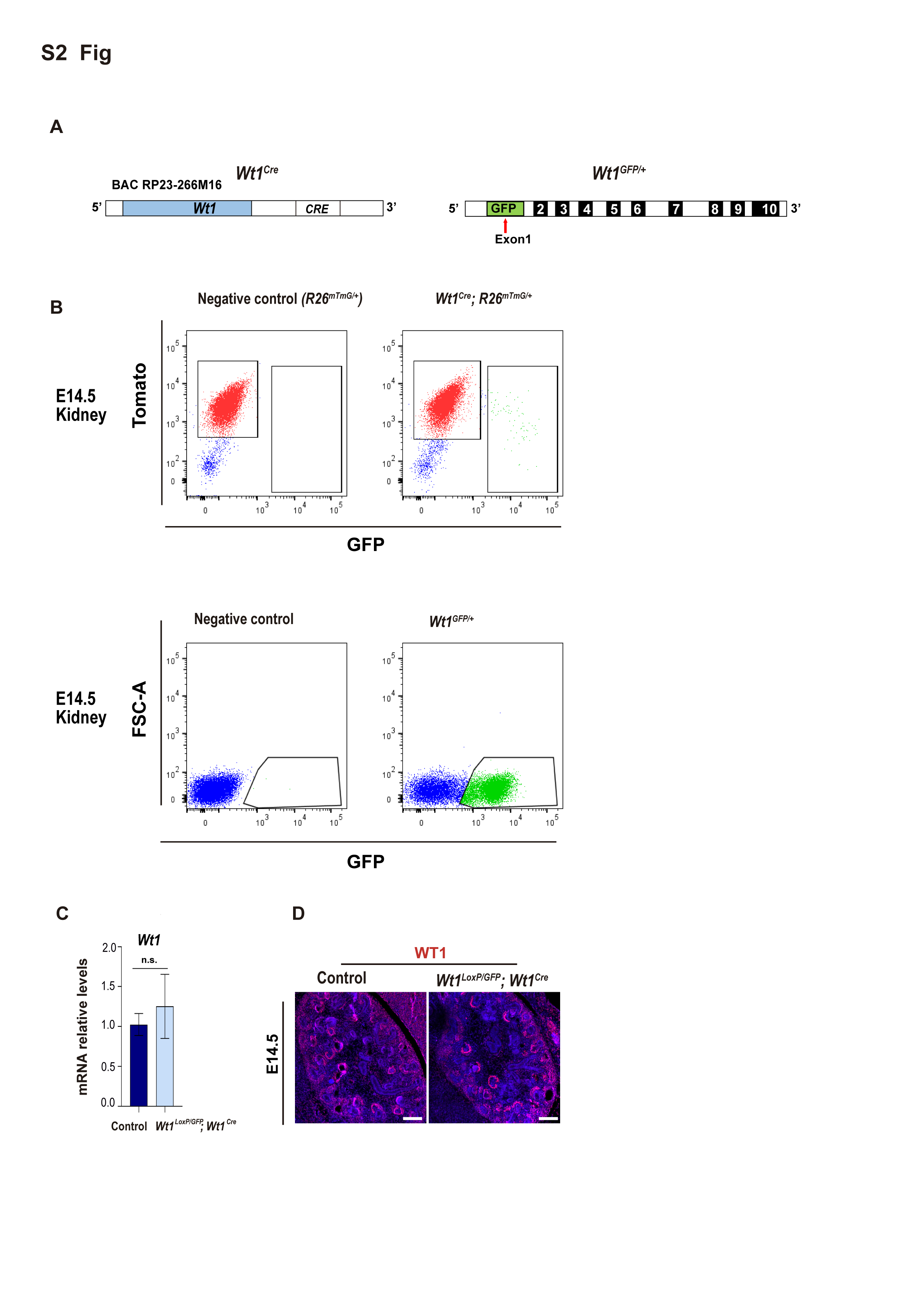

Supplement: S2 Fig — (A) Schematic representation of the transgenic constructs used to generate Wt1Cre and Wt1GFP/+ mice. (B) FACS analysis of the digested kidneys from E14.5 Wt1Cre;R26mTmG/+ and Wt1GFP/+ mice. Plots from negative-control littermate kidneys are also shown. Note the absence of GFP-positive cells in the kidneys of Wt1Cre;R26mTmG/+ mice. (C) qRT-PCR analysis of Wt1 in E14.5 kidneys from control and Wt1LoxP/GFP;Wt1Cre mice. Values represent the mean ± s.e.m. (n = 4–5). *P < 0.05, Student’s t-test. (D) Immunofluorescence staining for WT1 (red) and nuclear DAPI staining (blue), using kidney sections from E14.5 control and Wt1LoxP/GFP;Wt1Cre mice. Representative immunostaining images from a minimum of three control and mutant mice are shown. Scale bars: 100 μm. (TIF) [file pgen.1010240.s002.tif]

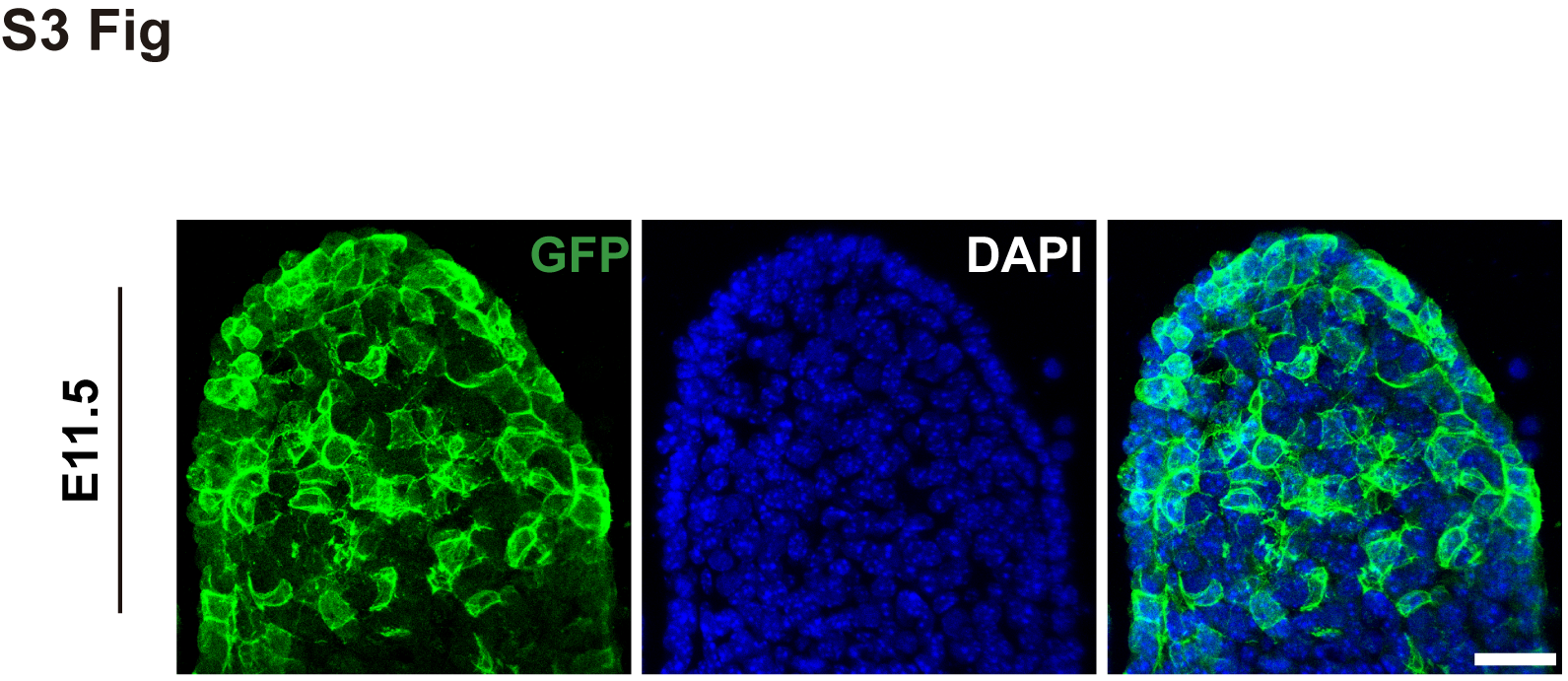

Supplement: S3 Fig — Immunofluorescence staining of GFP (green) and nuclear DAPI staining (blue), using sections from Wt1Cre;R26mTmG/+ E11.5 mice. GFP-positive cells were detected in the coelomic epithelium and somatic cells of the gonads. Representative immunostaining images from a minimum of three embryos are shown. Scale bar: 25μm. (TIF) [file pgen.1010240.s003.tif]

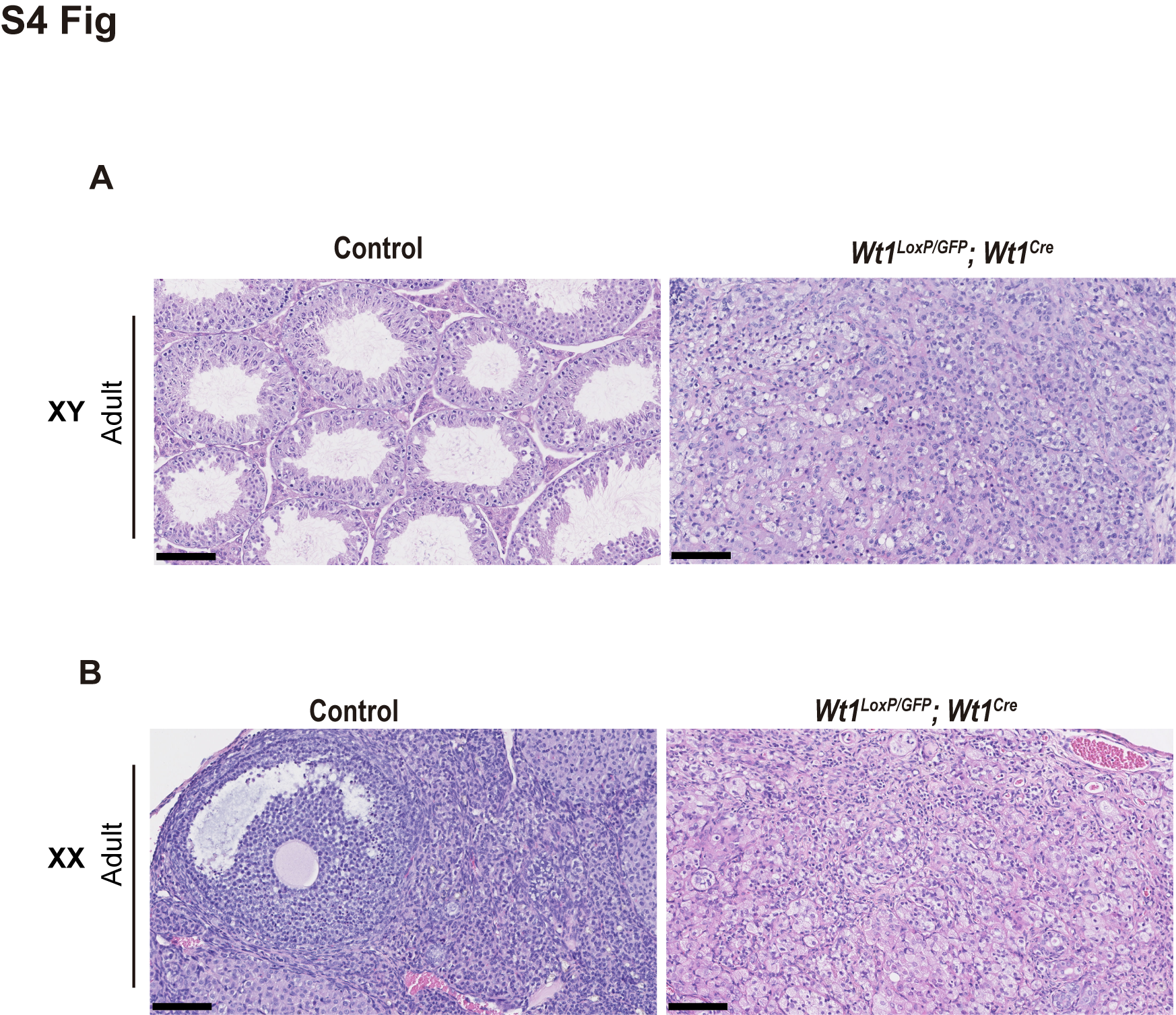

Supplement: S4 Fig — Higher magnification of the H&E staining of adult gonads from (A) XY and (B) XX control and Wt1LoxP/GFP;Wt1Cre mice included in Figs 3A and 4A. Note the presence of seminiferous tubules and follicles in the control testis and ovary and their absence in the gonads of Wt1LoxP/GFP;Wt1Cre mice. Images are representative of at least three each of XY and XX control and Wt1LoxP/GFP;Wt1Cre adult mice. Scale bars: 100 μm. (TIF) [file pgen.1010240.s004.tif]

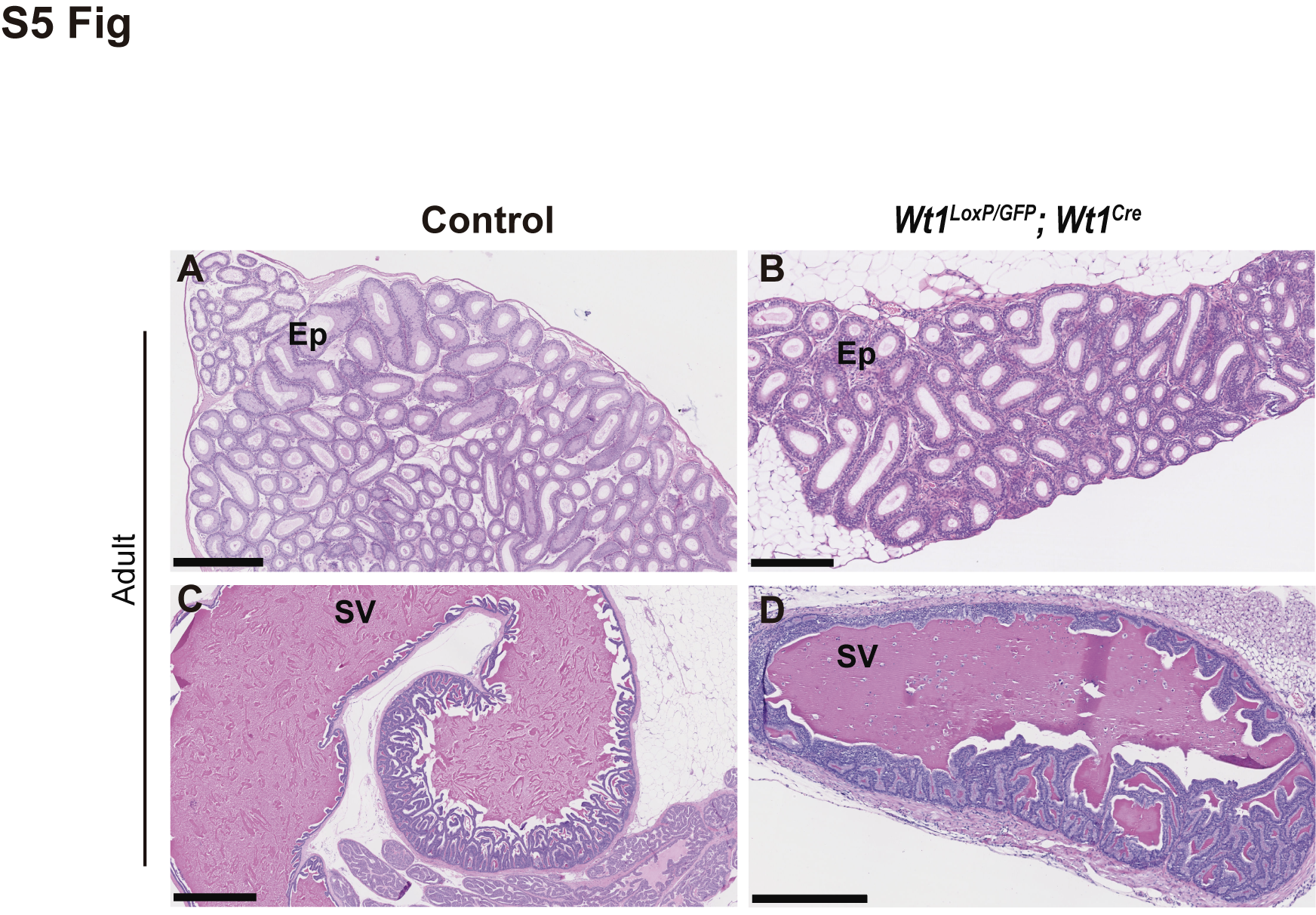

Supplement: S5 Fig — H&E staining of adult genital tract structures from XY control and Wt1LoxP/GFP;Wt1Cre mice. (A, C) Magnified images of Fig 3A (a, c). Images are representative of three of each control and Wt1LoxP/GFP;Wt1Cre adult mice. Scale bars: 500 μm in A,D; 250 μm in B and 1mm in C. (TIF) [file pgen.1010240.s005.tif]

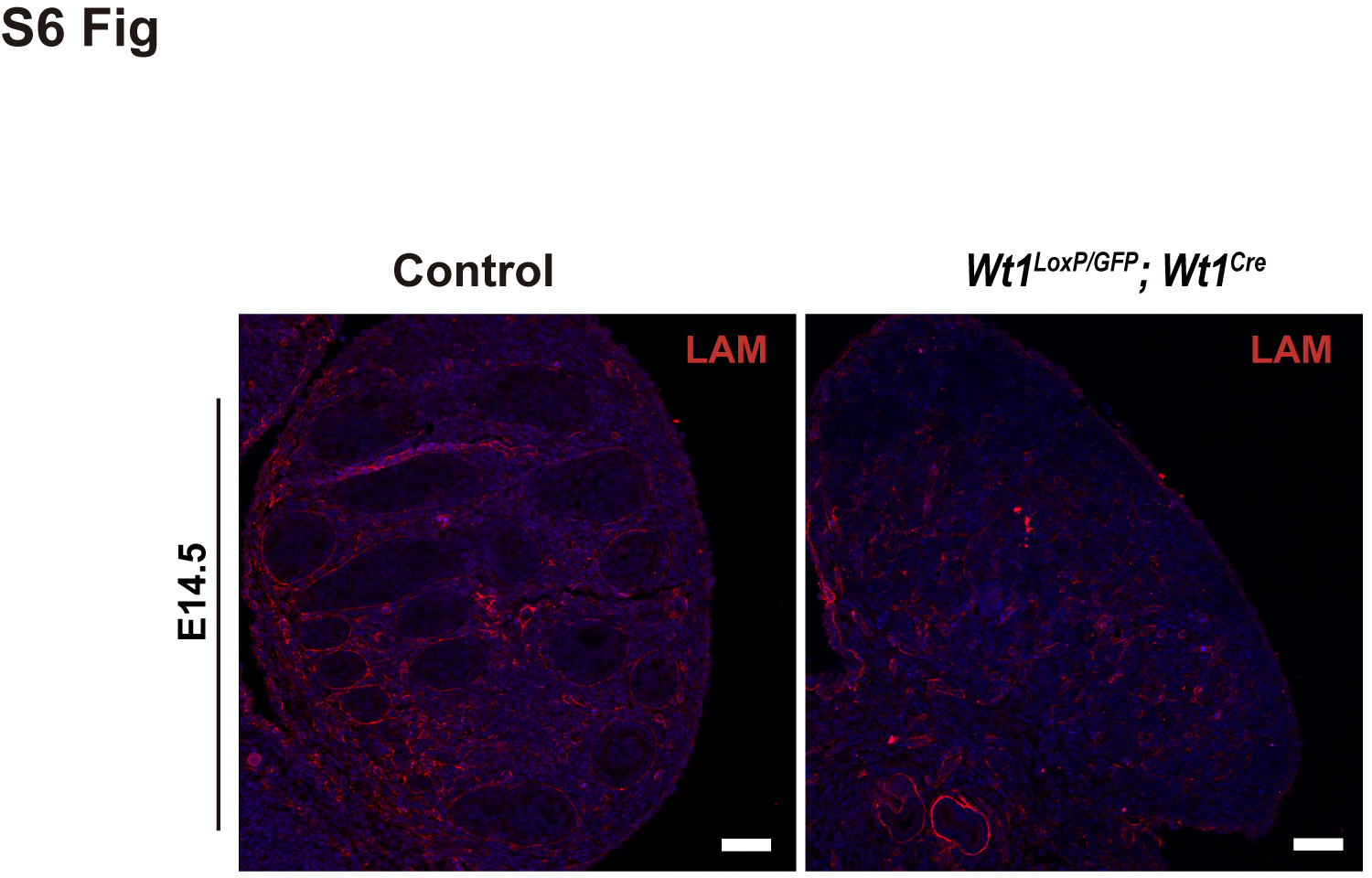

Supplement: S6 Fig — Immunofluorescence staining for laminin (LAM) (red) and nuclear DAPI staining (blue), using gonad sections from E14.5 XY control and Wt1LoxP/GFP;Wt1Cre mice. Representative immunostaining images from a minimum of three each of control and mutant mice are shown. Scale bars: 50 μm. (TIF) [file pgen.1010240.s006.tif]

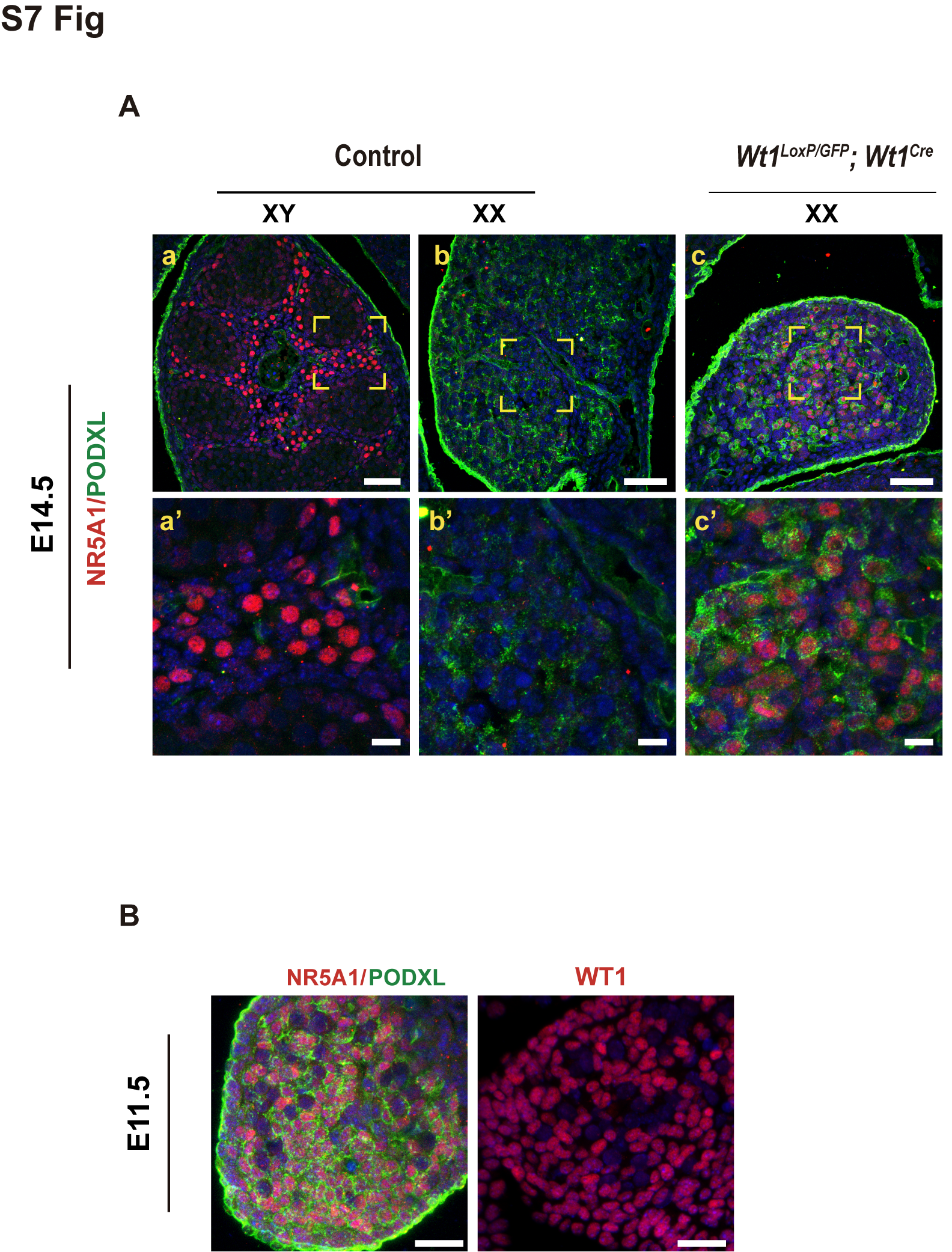

Supplement: S7 Fig — (A) Immunofluorescence staining for NR5A1 (red), podocalyxin (PODXL) (green) and nuclear DAPI staining (blue), using gonad sections from XY and XX control and XX Wt1LoxP/GFP;Wt1Cre E14.5 mice. (a’-c’) Magnified images of the corresponding boxed areas of the lower magnification. (B) Immunofluorescence staining for NR5A1 (red), PODXL (green) and nuclear DAPI staining (blue) and WT1 (red) and nuclear DAPI staining (blue), using gonad sections from XX control mice at E11.5. Note that the NR5A1-positive cells of the mutant gonads co-express PODXL similar to the XX early progenitor cells, while foetal Leydig NR5A1-positive cells are negative for PODXL. Representative immunostaining images from a minimum of three of each control and mutant mice are shown. Scale bars: 50 μm in a-c, 10 μm in a’-c’ and 25 μm in B. (TIF) [file pgen.1010240.s007.tif]
