# Supplementary material for: Deletion of Wt1 during early gonadogenesis leads to differences of sex development in male and female adult mice
Source: PLoS Genet. 2022 Jun 15;18(6):e1010240. doi: 10.1371/journal.pgen.1010240 (PMC9200307; doi:10.1371/journal.pgen.1010240)
Supplement: S1 Table — Distribution of the different genetic combinations resulting from the intercross of Wt1GFP/+;Wt1Cre males with Wt1LoxP/ LoxP females. (DOCX) [file pgen.1010240.s008.docx]

**S1 Table. Submendelian distribution of postnatal *Wt1^LoxP/GFP^;Wt1^Cre^*** **mice.**

|  | *Wt1^LoxP/+^;Wt1^Cre-^* | *Wt1^LoxP/+^;Wt1^Cre^* | *Wt1^LoxP/GFP^;Wt1^Cre-^* | ***Wt1^LoxP/GFP^;Wt1^Cre^*** |
| --- | --- | --- | --- | --- |
| E14.5 | 19/78  (24.36%) | 18/78  (23.78%) | 23/78  (29.49%) | 18/78  (23.08%) |
| P5 | 24/66  (36.36%) | 19/66  (28.79%) | 15/66  (22.73%) | 8/66  (12.12%) |
| 6 months | 24/82  (29.72%) | 26/82  (31.03%) | 20/82  (24.39%) | 12/82  (14.63%) |
