# Supplementary material for: Deletion of Wt1 during early gonadogenesis leads to differences of sex development in male and female adult mice
Source: PLoS Genet. 2022 Jun 15;18(6):e1010240. doi: 10.1371/journal.pgen.1010240 (PMC9200307; doi:10.1371/journal.pgen.1010240)
Supplement: S2 Table — (DOCX) [file pgen.1010240.s009.docx]

**S2 Table. Information of mouse models used in this article.**

| Transgenic mouse model | Reference | Additional information |
| --- | --- | --- |
| *Wt1^GFP/+^* | Hosen et al., 2007 [[1](#_ENREF_1)] | Mixed CD1 background |
| *Wt1^Cre^* | Wessels et al., 2012 [[2](#_ENREF_2)] | Mixed C57Bl/6J background |
| *Wt1^LoxP/LoxP^* | Martinez-Estrada et al., 2010 [[3](#_ENREF_3)] | Mixed CD1 background |
| *R26^mTmG/mTmG^* | Muzumdar et al., 2007 [[4](#_ENREF_4)] | Mixed C57Bl/6J background |
| *Wt1^LoxP/GFP^;Wt1^Cre^* | This article | Mixed background |

References

1. Hosen N, Shirakata T, Nishida S, Yanagihara M, Tsuboi A, Kawakami M, et al. The Wilms' tumor gene WT1-GFP knock-in mouse reveals the dynamic regulation of WT1 expression in normal and leukemic hematopoiesis. Leukemia. 2007;21(8):1783-91. doi: 10.1038/sj.leu.2404752. PubMed PMID: 17525726.

2. Wessels A, van den Hoff MJ, Adamo RF, Phelps AL, Lockhart MM, Sauls K, et al. Epicardially derived fibroblasts preferentially contribute to the parietal leaflets of the atrioventricular valves in the murine heart. Developmental biology. 2012;366(2):111-24. doi: 10.1016/j.ydbio.2012.04.020. PubMed PMID: 22546693; PubMed Central PMCID: PMC3358438.

3. Martinez-Estrada OM, Lettice LA, Essafi A, Guadix JA, Slight J, Velecela V, et al. Wt1 is required for cardiovascular progenitor cell formation through transcriptional control of Snail and E-cadherin. Nature genetics. 2010;42(1):89-93. doi: 10.1038/ng.494. PubMed PMID: 20023660; PubMed Central PMCID: PMC2799392.

4. Muzumdar MD, Tasic B, Miyamichi K, Li L, Luo L. A global double-fluorescent Cre reporter mouse. Genesis. 2007;45(9):593-605. doi: 10.1002/dvg.20335. PubMed PMID: 17868096.
