# Supplementary material for: Deletion of Wt1 during early gonadogenesis leads to differences of sex development in male and female adult mice
Source: PLoS Genet. 2022 Jun 15;18(6):e1010240. doi: 10.1371/journal.pgen.1010240 (PMC9200307; doi:10.1371/journal.pgen.1010240)
Supplement: S3 Table — (DOCX) [file pgen.1010240.s010.docx]

**S3 Table. List of primers used for genotyping.**

| Gene | Sequence |
| --- | --- |
| *Cre/F* | 5’-GCATTACCGGTCGATGCAACGAGTGATGAG-3’ |
| *Cre/R* | 5’-GAGTGAACGAACCTGGTCGAAATCAGTGCG-3’ |
| *Fabpi200/F* | 5’-TGGACAGGACTGGACCTCTGCTTTCCTAGA-3’ |
| *Fapbi200/R* | 5’-TAGAGCTTTGCCACATCACAGGTCATTCAG-3’ |
| *Wt1^loxP^/F* | 5’-TGGGTTCCAACCGTACCAAAGA-3’ |
| *Wt1^loxP^/R* | 5’-GGGCTTATCTCCTCCCATGT-3’ |
| *Wt1^loxP^/R2* | 5’-GTACGCGCGAACACTGACTA-3’ |
| *Wt1^GFPm^/F* | 5’-GCCTGAAGAACGAGATCAGC-3’ |
| *Wt1^GFPm^/F2* | 5’-AGCCTGAAGCTGCTCACATCC-3’ |
| *Wt1^GFPm^/R* | 5’-GGCAGCTTGAATTCCTCTCA-3’ |
| *Rbm31 F* | 5'-CACCTTAAGAACAAGCCAATACA-3' |
| *Rbm31 R* | 5'-GGCTTGTCCTGAAAACATTTGG-3' |
| *R26g2F* | 5'-TGTTATCAGTAAGGGAGCT-3' |
| *R26g2/Rmut* | 5'-AAGACCGCGAAGAGTTTGT-3' |
| *R26g2/Rwt* | 5'-CACACCAGGTTAGCCTTTA-3' |
