# Supplementary material for: Deletion of Wt1 during early gonadogenesis leads to differences of sex development in male and female adult mice
Source: PLoS Genet. 2022 Jun 15;18(6):e1010240. doi: 10.1371/journal.pgen.1010240 (PMC9200307; doi:10.1371/journal.pgen.1010240)
Supplement: S4 Table — (DOCX) [file pgen.1010240.s011.docx]

**S4 Table. List of antibodies used in this study.**

| Antibody | Dilution | Description | Manufacturer |
| --- | --- | --- | --- |
| WT1 | 1:100 | Rabbit | Abcam ab89901 |
| GFP | 1:300 | Goat | Abcam ab6673 |
| SOX9 | 1:100 | Rabbit | Millipore AB5535 |
| FOXL2 | 1:100 | Goat | Novus Biologicals NB100-1277 |
| DDX4 | 1:200 | Rabbit | Abcam ab13840 |
| NR5A1 | 1:200 | Rabbit | Proteintech 18658-1-AP |
| LAMININ | 1:300 | Rabbit | Sigma L9393 |
| αSMA | 1:300 | Rabbit | Cell signaling 19245 |
| CDH1 | 1:100 | Mouse | BD Biosciences 610181 |
| PODXL | 1:200 | Goat | R&D Systems AF1556 |
| PCNA | 1:150 | Mouse | Santa Cruz sc-25280 |
| RALDH2 | 1:300 | Rabbit | Sigma ABN420 |
