# Supplementary material for: Deletion of Wt1 during early gonadogenesis leads to differences of sex development in male and female adult mice
Source: PLoS Genet. 2022 Jun 15;18(6):e1010240. doi: 10.1371/journal.pgen.1010240 (PMC9200307; doi:10.1371/journal.pgen.1010240)
Supplement: S5 Table — (DOCX) [file pgen.1010240.s012.docx]

**S5 Table. List of primers used in the quantitative real-time PCR analysis.**

|  | **Primer** | **Sequence 5'-3'** |
| --- | --- | --- |
| *Wt1* | F | TTCAAGGACTGCGAGAGAAG |
|  | R | GGGAAAACTTTCGCTGACAA |
| *Wnt4* | F | AGACGTGCGAGAAACTCAAAG |
|  | R | GGAACTGGTATTGGCACTCCT |
| *Sox9* | F | CAGCAAGACTCTGGGCAAG |
|  | R | TCCACGAAGGGTCTCTTCTC |
| *Sry* | F | GCTGGGATGCAGGTGGAAAA |
|  | R | CCCTCCGATGAGGCTGATATT |
| *Fgf9* | F | GGGGAGCTGTATGGATCAGA |
|  | R | TCCCGTCCTTATTTAATGCAA |
| *Rspo1* | F | CGACATGAACAAATGCATCA |
|  | R | CTCCTGACACTTGGTGCAGA |
| *Podxl* | F | TCCTTGTTGCTGCCCTCT |
|  | R | CTCTGTGAGCCGTTGCTG |
| *18S* | F | CGATTGGATGGTTTAGTGAGG |
|  | R | AGTTCGACCGTCTTCTCAGC |
| *Fst* | F | TGGATTAGCCTATGAGGGAAAG |
|  | R | TGGAATCCCATAGGCATTTT |
| *Amh* | F | CCACACCTCTCTCCACTGGTA |
|  | R | GGCACAAAGGTTCAGGGGG |
| *Runx1* | F | GCAGGCAACGATGAAAACTACT |
|  | R | GCAACTTGTGGCGGATTTGTA |
| *Foxl2* | F | ACAACACCGGAGAAACCAGAC |
|  | R | CGTAGAACGGGAACTTGGCTA |
| *Hsd3b1* | F | CTCAGTTCTTAGGCTTCAGCAATTAC |
|  | R | CCAAAGGCAAGATATGATTTAGGA |
| *Star* | F | CCGGAGCAGAGTGGTGTCA |
|  | R | CAGTGGATGAAGCACCATGC |
| *Cyp11a1* | F | CCAGTGTCCCCATGCTCAAC |
|  | R | TGCAGTGTCCTTCCAGGTCT |
| *Stra8* | F | CTCCTCCTCCACTCTGTTGC |
|  | R | GCGGCAGAGACAATAGGAAG |
| *Dppa3* | F | GACCCAATGAAGGACCCTGAA |
|  | R | GCTTGACACCGGGGTTTAG |
| *Nanog* | F | AGGCTTTGGAGACAGTGAGGTG |
|  | R | TGGGTAAGGGTGTTCAAGCACT |
